# Light-strand bias and enriched zones of embedded ribonucleotides are associated with DNA replication and transcription in the human-mitochondrial genome

**DOI:** 10.1093/nar/gkad1204

**Published:** 2023-12-20

**Authors:** Penghao Xu, Taehwan Yang, Deepali L Kundnani, Mo Sun, Stefania Marsili, Alli L Gombolay, Youngkyu Jeon, Gary Newnam, Sathya Balachander, Veronica Bazzani, Umberto Baccarani, Vivian S Park, Sijia Tao, Adriana Lori, Raymond F Schinazi, Baek Kim, Zachary F Pursell, Gianluca Tell, Carlo Vascotto, Francesca Storici

**Affiliations:** School of Biological Sciences, Georgia Institute of Technology, Atlanta 30332, GA, USA; School of Biological Sciences, Georgia Institute of Technology, Atlanta 30332, GA, USA; School of Biological Sciences, Georgia Institute of Technology, Atlanta 30332, GA, USA; School of Biological Sciences, Georgia Institute of Technology, Atlanta 30332, GA, USA; School of Biological Sciences, Georgia Institute of Technology, Atlanta 30332, GA, USA; School of Biological Sciences, Georgia Institute of Technology, Atlanta 30332, GA, USA; School of Biological Sciences, Georgia Institute of Technology, Atlanta 30332, GA, USA; School of Biological Sciences, Georgia Institute of Technology, Atlanta 30332, GA, USA; School of Biological Sciences, Georgia Institute of Technology, Atlanta 30332, GA, USA; Department of Medicine, University of Udine, Udine 33100, Italy; IMol Polish Academy of Sciences, Warsaw 02-247, Poland; Department of Medicine, University of Udine, Udine 33100, Italy; General Surgery Clinic and Liver Transplant Center, University-Hospital of Udine, Udine 33100, Italy; Department of Biochemistry and Molecular Biology, Tulane Cancer Center, Tulane University of Medicine, New Orleans, LA 70118, USA; Center for ViroScience and Cure, Department of Pediatrics, Laboratory of Biochemical Pharmacology, Emory University School of Medicine and Children’s Healthcare of Atlanta, Atlanta 30322, GA, USA; Department of Psychiatry and Behavioral Sciences, Emory University, Atlanta 30329, GA, USA; Department of Population Science, American Cancer Society, Kennesaw 30144, GA, USA; Center for ViroScience and Cure, Department of Pediatrics, Laboratory of Biochemical Pharmacology, Emory University School of Medicine and Children’s Healthcare of Atlanta, Atlanta 30322, GA, USA; Center for ViroScience and Cure, Department of Pediatrics, Laboratory of Biochemical Pharmacology, Emory University School of Medicine and Children’s Healthcare of Atlanta, Atlanta 30322, GA, USA; Department of Biochemistry and Molecular Biology, Tulane Cancer Center, Tulane University of Medicine, New Orleans, LA 70118, USA; Laboratory of Molecular Biology and DNA Repair, Department of Medicine, University of Udine, Udine 33100, Italy; Department of Medicine, University of Udine, Udine 33100, Italy; IMol Polish Academy of Sciences, Warsaw 02-247, Poland; School of Biological Sciences, Georgia Institute of Technology, Atlanta 30332, GA, USA

## Abstract

Abundant ribonucleoside-triphosphate (rNTP) incorporation into DNA by DNA polymerases in the form of ribonucleoside monophosphates (rNMPs) is a widespread phenomenon in nature, resulting in DNA-structural change and genome instability. The rNMP distribution, characteristics, hotspots and association with DNA metabolic processes in human mitochondrial DNA (hmtDNA) remain mostly unknown. Here, we utilize the *ribose-seq* technique to capture embedded rNMPs in hmtDNA of six different cell types. In most cell types, the rNMPs are preferentially embedded on the light strand of hmtDNA with a strong bias towards rCMPs; while in the liver-tissue cells, the rNMPs are predominately found on the heavy strand. We uncover common rNMP hotspots and conserved rNMP-enriched zones across the entire hmtDNA, including in the control region, which links the rNMP presence to the frequent hmtDNA replication-failure events. We show a strong correlation between coding-sequence size and rNMP-embedment frequency per nucleotide on the non-template, light strand in all cell types, supporting the presence of transient RNA-DNA hybrids preceding light-strand replication. Moreover, we detect rNMP-embedment patterns that are only partly conserved across the different cell types and are distinct from those found in yeast mtDNA. The study opens new research directions to understand the biology of hmtDNA and genomic rNMPs.

## Graphical Abstract

**Graphical Abstract:**
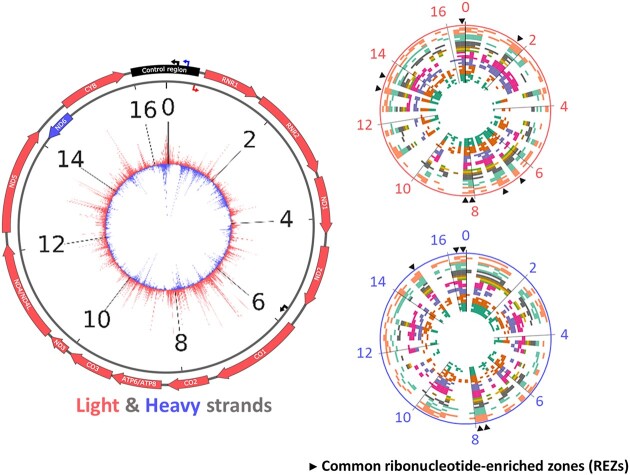

## Introduction

Human-mitochondrial DNA (hmtDNA) consists of two strands and 37 genes, including 13 coding sequences (CDSs), 2 ribosomal RNA (rRNA) genes and 22 transfer RNA (tRNA) genes (1). Maintaining mtDNA functions is essential to human cells. Malfunction of the hmtDNA-replication machinery leads to mtDNA-depletion syndromes or mtDNA-deletion disorders, resulting in respiratory-chain deficiency and neuromuscular symptoms in patients (2). Ribonucleoside triphosphates (rNTPs) are the basic building blocks of RNA. Due to their structural similarity to deoxyribonucleoside triphosphates (dNTPs), rNTPs are abundantly incorporated into DNA in the form of ribonucleoside monophosphates (rNMPs) by replicative DNA polymerases (Pols), despite DNA-Pol ability to differentiate the sugar moieties (3–5). The incorporated rNMPs constitute the most abundant non-standard nucleotides (nt) in the DNA of eukaryotic and prokaryotic genomes (6–9). The rNMP-embedment rates of replicative DNA Pols, including DNA Pol α, δ, ϵ for eukaryotic nuclear DNA, and Pol γ for mtDNA have been studied *in vitro* (7,10–12). At normal dNTP concentrations, human Pol γ incorporates approximately one rNMP per ∼2300 nt (12,13). The majority of the rNMPs embedded in nuclear DNA are efficiently removed through the ribonucleotide-excision repair (RER) pathway, which is initiated by ribonuclease (RNase) H2 (14). Inactivation of RER can be caused by mutations in any of the three RNase H2 subunits (15). Deficiency in rNMP removal from nuclear DNA is associated with human diseases such as Aicardi-Goutières Syndrome (AGS), Systemic Lupus Erythematosus (SLE), and different types of cancer (7,16–18). In yeast and human mtDNA, the embedded rNMPs cannot be repaired by RER due to the absence of RNase H2 in the organelle (19–21). As a result, mtDNA has abundant rNMPs (21,22). The presence of rNMPs can cause DNA fragility and structural change and affect DNA-protein interactions in nuclear DNA (23,24). Although human DNA Pol γ can efficiently bypass single rNMPs with high fidelity (13), work in mouse cells has shown that high-level of rGMP incorporation induced by deficiency of the mitochondrial inner membrane protein MPV17 impairs mouse mtDNA replication and results in pathology (18).

Only recently, different rNMP-mapping techniques have been developed including ribose-seq (22), emRiboSeq (25), HydEn-seq (26), Pu-seq (27), RiSQ-seq (28), and GLOE-Seq (29). These techniques can capture the embedded rNMPs in genomic DNA and facilitate genomic studies on rNMPs. Bioinformatic tools have also been developed to map the rNMPs in genomic DNA (30), optimize the rNMP capture techniques (31), and analyze rNMP-embedment characteristics (32,33). Many of the rNMP-embedment characteristics in mtDNA of the yeast *Saccharomyces cerevisiae*, *Saccharomyces paradoxus*, *Schizosaccharomyces pombe* and the green alga *Chlamydomonas reinhardtii* have been illustrated in detail (21,34). However, current knowledge about rNMP embedment in hmtDNA has been limited to count and composition in HeLa and fibroblast cells (12). It remains unknown whether the rNMPs found in hmtDNA have a biased distribution, whether rNMP hotspots and patterns exist in hmtDNA, and whether these rNMP features differ in distinct human cell types and across species and are associated with specific hmtDNA metabolic functions. Uncovering the specific characteristics of rNMPs found in hmtDNA can reveal the relation between rNMPs in DNA and many human diseases associated with the alteration of mtDNA metabolism and stability.

In this study, we analyze 32 hmtDNA libraries of six different human cell types, which we constructed using the ribose-seq technique. Our results identify strand bias, hotspots, and preferred patterns of rNMPs in hmtDNA. Furthermore, we uncover the presence of rNMP-enriched zones (REZs), including within the control region. We discuss the potential cause/effect of these rNMP features concerning hmtDNA replication and transcription. In summary, our study unveils an unexpected relationship between the embedded rNMPs, mtDNA replication, transcription, and coding sequences in the hmtDNA genome.

## Materials and methods

### Human cell line preparation

*Human primary activated CD4^+^ T cells*. CD4^+^T cells were isolated from human buffy coats of five health donors (NY Blood Center) as previously described (35), and the isolated CD4^+^T cells were pooled and activated by phytohemagglutinin (5 ng/ml) and IL-2 (5 ng/ml) for 3 days. The total cellular DNAs of the activated CD4 + T cells were isolated using a DNA extraction kit (Promega Wizard) for analysis.

*hESC-H9*. Human Embryonic Stem Cells (hESC) line H9, passage 22, were purchased from WiCell Research Institute, University of Wisconsin (line WA09, lot # WB66595). hESC was maintained and expanded in feeder-free culture conditions with mTeSR basal medium (STEMCELL Technologies Inc., Vancouver, Canada, cat # 85850) on 6-well plates culture-treated coated with Corning Matrigel hESC-qualified Matrix, LDEV free (Corning Inc., Corning, NY, USA, catalog # 35427) in a humidified chamber in a 5% CO_2_-air mixture at 37°C. The culture medium was changed daily, and regions of differentiation were removed by aspiration. Cells were passaged as small aggregates every four to five days at ratios of 1:3 to 1:6 using a gentle dissociation reagent (Corning Inc., cat # 3010). Cells were harvested at passage 28 as a single-cell suspension for downstream applications.

*DLTB and TLTB*. Paired HCC Tumor Liver Tissue Biopsy (TLTB) and adjacent non-tumor Distal Liver Tissue Biopsy (DLTB) from patients undergoing HCC resection were obtained from the Department of Medicine, General Surgery and Transplantation of the University of Udine, Udine, Italy. None of the patients had received any local or systemic anticancer treatments before the surgery. Both tumor and non-tumor tissues were histologically confirmed. Mitochondria were isolated from surgical biopsies as reported by Bazzani *et al.* (36). Then, mtDNA was extracted and purified using Qiagen genomic-tip 20/G and following the manufacturer's indications. DLTB and TLTB samples of 17 patients affected by HCC and characterized by the cirrhotic liver due to alcohol abuse or HCV and HBV infection were pooled together to obtain a sufficient amount of mtDNA to construct the ribose-seq library. The individual DLTB-8 and TLTB-8 samples were obtained from a 68-year-old Caucasian male, who was diagnosed with a large HCC located in the right liver lobe during a routine ultrasound. A computed tomography scan confirmed the diagnosis. Differently from the pooled sample, the patient had no history of alcohol intake and was negative for HCV and HBV. Liver function was normal without signs and symptoms of portal hypertension (Child-Pugh score A-6 and MELD 7). His preoperative alpha-fetoprotein (AFP) was 4.8 ng/ml. He underwent an eventful open right hepatectomy in 2015; during surgery, the liver was macroscopically normal without cirrhosis. Pathological examination revealed a well-differentiated HCC (G1) with a maximum diameter of 9.5 cm, microvascular invasion was absent resulting in a pathological staging pT1, N0, M0. The patient has been followed up at the Institution yearly since the last follow-up in April 2022 when he resulted negative for HCC recurrence, with normal liver function and an AFP of 4.6 ng/ml.

*WB-GTP*. Whole Blood Grady Trauma Project (WB-GTP) was part of a larger investigation of genetic and environmental factors in a predominantly African American (AA) urban population of low socioeconomic status to determine how those factors may modulate the response to stressful life events. Research participants were approached in the waiting rooms of primary care of a large, public hospital (Grady Memorial Hospital in Atlanta, Georgia) while either waiting for their medical appointments or while waiting with others who were scheduled for medical appointments. Screening interviews, including the participants’ demographic information (e.g. self-identified race, sex and age) and psychiatric history were completed on-site, including the Clinician Administered PTSD Scale (CAPS (37)). Subjects were scored as having PTSD if they met DSM-IV PTSD criteria from the CAPS interview. Exclusion criteria included mental retardation or active psychosis. Further details regarding the Grady Trauma Project (GTP) dataset can be found in Gillespie *et al.* (38). Written and verbal informed consent was obtained for all subjects. DNA from the participant's blood was extracted using either the E.Z.N.A. Mag-Bind Blood DNA Kit (Omega Bio-Tek, Inc., Norcross, GA) or ArchivePure DNA Blood Kit (5 Prime, Inc., Gaithersburg, MD) following protocol instructions. Matching samples with the highest yield were selected for the analyses. All procedures in this study were approved by the Institutional Review Boards of Emory University School of Medicine and Grady Memorial Hospital.

*HCT116*. HCT116 cells were provided by the Pursell group at Tulane University. The cells were grown in Dulbecco's modification of Eagle's medium (DMEM) containing non-essential amino acids (Corning) with 10% fetal bovine serum (Sigma-Aldrich) and 1x penicillin-streptomycin (Gibco). Cells were grown at 37°C in a 5% CO_2_-humified incubator. Cells were removed from the plate by 3–5 min incubation in media containing trypsin. Cells were spun down and the supernatant was removed. Cells were lysed by the addition of TNES lysis buffer containing Proteinase K followed by phenol: chloroform extraction. Genomic DNA was ethanol precipitated, air dried, and concentration quantified using a Nanodrop and Qubit fluorometer (Thermo Fisher Scientific).

*HEK-293T*. Human embryonic kidney T (HEK-293T) RNASEH2A wild-type and KO (RNH2A-KO) cells were provided by the Pursell group at Tulane University. The cells were grown in Dulbecco's modification of Eagle's medium (DMEM) containing 4.5 g/l glucose, l-glutamine, and sodium pyruvate (Corning) with 10% fetal bovine serum (Sigma-Aldrich) and 1× penicillin–streptomycin (Gibco). Cells were grown at 37°C in a 5% CO_2_-humified incubator. For the construction of the RNH2A KO clones, RNASEH2A (Chr19, exon 2) gRNA (5′-TAACAGATGGCGTAGACCAT-3′) was cloned into GeneArtTM CRISPR Nuclease Vector with OFP reporter (Invitrogen) following manufacturer's protocol. HEK-293T cells were transfected with 6 mL of Lipofectamine 2000 (Invitrogen) and 2.5 mg of gRNA vector DNA when the cells were 60–70% confluent. After 48 hours, OFP-positive cells were FACs sorted and serially diluted into 96-well plates at ∼1.5 cells per well and incubated for 10–14 days. HEK-293T RNH2A-KO T3-8 and T3-17 have three distinct frameshift mutations consistent with all three alleles being modified in hypotriploid 293T cells, respectively. The RNH2A-KO T3-8 has an insertion of G at position −1; deletion of five bases at position +2 to +6; complex alteration with deletion of three bases from position −1 to +2, deletion of two bases from position +5 to +6, and CC > TT at position +8 and +9. The RNH2A-KO T3-17 has the deletion of 14 bases from position +7 to −7; the deletion of six bases from position −5 to 1; and the deletion of 25 bases from position −3 to +22. All positions are indicated with respect to the Cas9 cleavage site on the reference strand.

### Ribose-seq library preparation

The libraries in this study were prepared using the latest ribose-seq method that has been upgraded from the previous method (21,22,39). To construct libraries with human cell lines, we improved the ribose-seq protocol by (i) introducing NEBNext® dsDNA Fragmentase® (New England Biolabs) to fully fragment the human genomic DNA samples; (ii) optimizing the removal of linear ssDNA process to retain more rNMPs; (iii) applying a new size selection method using HighPrep™ PCR Clean-up System (MagBio Genomics) to increase the yield of captured rNMPs.

All the commercial enzymes applied in the ribose-seq protocol were used according to the manufacturer's recommended instructions. Genomic DNA samples were fragmented using NEBNext® dsDNA Fragmentase® or a combination of restriction enzymes to generate small DNA fragments. Various sets of restriction enzymes were applied to fragment human genomic DNA samples, as indicated in t. The used combinations were (i) RE1: [HpyCH4V] + [Hpy116II, Eco53KI, RsaI and StuI]; (ii) RE2: [AleI, AluI and PvuII] + [DraI, HaeIII and SspI]; (iii) RE3: [CviKl-1] + [Mly, MscI and MslI].

Following fragmentation with restriction endonucleases, the fragmented DNA was purified by QIAquick PCR Purification Kit (Qiagen). The fragments were tailed with dATP (New England Biolabs) by using Klenow Fragment (3′→5′ exo-) (New England Biolabs) for 30 min at 37°C and purified by using a QIAquick PCR Purification Kit. In case of fragmentation with NEBNext® dsDNA Fragmentase®, NEBNext End Repair Module (New England Biolabs) was performed before dA-tailing to convert fragmented DNA to blunt-ended DNA having 5′ phosphates and 3′-hydroxyls.

Following dA-tailing and purification, a partially double-stranded adapter (Adapter.L1 - Adapter.L8 with Adapter.S (21), Supplementary Table S1) was annealed with the DNA fragments by T4 DNA ligase (New England Biolabs) incubating overnight at 16°C. Following overnight ligation, the products were purified using the HighPrep™ PCR Clean-up System.

The adapter-annealed fragments were then treated with 0.3 M NaOH (Working concentration) for 2 h at 55°C to denature the DNA strands and cleave the 3′ site of embedded rNMP sites resulting in 2′,3′-cyclic phosphate, and 2′-phosphate termini.

After the treatment with Alkali, neutralization using 2 M HCl and purification using HighPrep™ RNA Elite Clean-up System (MagBio Genomics) was performed. Further purification steps were performed using HighPrep™ RNA Elite Clean-up System, except for the size selection step.

The single-stranded DNA (ssDNA) fragments were then incubated with 1 μM *Arabidopsis thaliana* tRNA ligase (AtRNL), 50 mM Tris–HCl pH 7.5, 40 mM NaCl, 5 mM MgCl_2_, 1 mM DTT, and 300 μM ATP for 1 h at 30°C to ligate the 5′-phosphate and 3′-OH of ssDNA fragments containing an rNMP, followed by bead purification.

The ssDNA fragments were treated with T5 Exonuclease (New England Biolabs) for 30 min at 37°C to degrade the unligated linear ssDNA fragments. After purification, the circular fragments were incubated with 1 μM 2′-phosphotransferase (Tpt1), 20 mM Tris–HCl pH 7.5, 5 mM

MgCl_2_, 0.1 mM DTT, 0.4% Triton X-100 and 10 mM NAD^+^ for 1 h at 30°C to remove the 2′-phosphate at the ligation junction.

Following purification, the circular ssDNA fragments were amplified with two steps of PCR and became a library. Both PCR began with an initial denaturation at 98°C for 30 s. Then denaturation at 98°C for 10 s, primer annealing at 65°C for 30 s, and DNA extension at 72°C for 30 s were done. These PCR steps were performed for 6 cycles in the first PCR round and for 11 cycles in the second PCR round. Lastly, there is a final extension reaction at 72°C for 2 min for both PCRs.

A first round of PCR was performed to extend the sequences of the Illumina adapter for TruSeq CD Index primers. The primers (PCR.1 and PCR.2) used for the first round were the same for all libraries. A second round of PCR was performed to insert specific indexes i7 and i5 into each library (21). Both PCR rounds were performed with Q5-High Fidelity polymerase (New England Biolabs) for 6 and 11 cycles, respectively.

Following the PCR cycles, DNA fragments between 250 and 700 bp were purified using the HighPrep™ PCR Clean-up System. The resulting ribose-seq libraries were mixed at equimolar concentrations and normalized to 4 nM. The libraries were sequenced on an Illumina Next 500 or HiSeq X Ten in the Molecular Evolution Core Facility at the Georgia Institute of Technology or Admera Health.

### Locate rNMPs in DNA

Raw sequenced reads are trimmed using Trim_galore software with the command ‘trim_galore -a AGTTGCGACACGGATCTATCA -q 15 –length 62 input.fastq -o output.fastq’. Using this command, the remaining adapter sequences and low-quality bases with sequencing quality scores lower than 15 are trimmed. Afterward, the Ribose-Map bioinformatics toolkit is used to map the incorporated rNMPs to the human genome GRCh38 mitochondrial DNA sequence (chrM) on a single-nucleotide level (30,40). Since Ribose-Map processes hmtDNA as a linear DNA, we need to recover the rNMP embedment around the zero position. Specifically, we used Ribose-Map to align the rNMPs to generate control-region rNMPs. Then, control-region rNMPs and linear hmtDNA rNMPs are merged to form the actual rNMPs in circular hmtDNA. Afterward, the background noise of RE-recognition sites is removed using the same protocol in (21). Some rNMPs mismatch the nucleotide at their alignment locations in the reference genome. These rNMPs are likely to be induced by sequencing error and are also removed from all libraries using customized python3 scripts available under GNU GPL V3.0 License on Zenodo (https://doi.org/10.5281/zenodo.10211459).

### Calculate the normalized frequencies for rNMP embedment

We use the rNMP-embedment probability per base (PPB) and enrichment factor to measure the rNMP-embedment frequency in a particular region. The PPB represents the rNMP-embedment probability at each nucleotide, which is comparable among the different genomic regions and different rNMP libraries. For genomic region *G* in library *L*, the PPB can be calculated using the following formula.


\begin{equation*}PP{B}_{G,L} = \frac{{{R}_{G,L}}}{{Length\left( G \right) \times {R}_{total,L}{\mathrm{\ }}}}\end{equation*}

${R}_{G,L}$ denotes the rNMP count in the genomic region *G* in library *L*, and ${R}_{total,L}$ denotes the total rNMP count in mtDNA in library *L*. The raw PPB value is used to measure the rNMP embedment frequency in coding sequence comparison. The moving average of PPB (window size = 51 nt) is used to measure the rNMP embedment frequency in the control region.

### The enrichment factor is similar to PPB but measures the rNMP embedment frequency in a region, which can be calculated as


\begin{equation*}E{F}_{G,L} = \frac{{{R}_{G,L}}}{{{R}_{total,{\mathrm{\ }}L}}} \times \frac{{Length\left( {Genome} \right)}}{{Length\left( G \right)}}\end{equation*}

The normalized frequency of the rNMP composition and rNMP patterns is calculated as described in Balachander *et al.* (21). Briefly, the count of rNMPs, dinucleotides, and trinucleotides are divided by the corresponding background dNMP count in the reference genome to get the frequency. For composition, the frequencies are further divided by the sum of frequencies to get the normalized frequencies. For dinucleotide and trinucleotide patterns, the frequencies are divided by the sum of frequencies that share the same rNMP to get the normalized frequencies. The normalized frequency is calculated using the RibosePreferenceAnalysis package (33).

### Determine the rNMP hotspots and high-frequency rNMP locations in mtDNA

To identify the rNMP hotspots for a specific cell type or all cell types combined, we searched the reference genome to find the single-nucleotide locations with incorporated rNMPs in all libraries using a customized R script. To identify high-frequency rNMP locations, which are the locations with the highest rNMP incorporated in a single base of hmtDNA, we calculated the frequency of rNMP counts at single-base locations for every library at each location on the chromosome with the presence of at least one rNMP. We then filtered out the top 1% locations with the most abundant embedded rNMPs and extracted ±3 bp genomic sequences around the rNMP locations to generate a consensus sequence using the ggseqlogo R package.

### Detect rNMP enriched zones (REZs) in hmtDNA

Both strands of hmtDNA were divided into 200-nt bins (*N* = 166). For each rNMP library, the rNMP enrichment factors (EF) were calculated in each bin. All bins with EF > 1 represent enriched zones in the library. The common REZs are the bins that are enriched in all cell categories and at least in 80% of the libraries.

### Background coverage calculation for different fragmentation Methods

Paired-end sequencing was performed on fragmented CD4^+^T cell DNA using restriction enzymes and/or dsDNA fragmentase. Paired-end sequencing reads were aligned to circular hmtDNA with a maximum insert size of 1000 nt. Afterward, coverage at each nucleotide in hmtDNA was calculated by piling up the aligned reads. The enrichment factor of coverage inside each 200-nt bin was calculated as background coverage.

### Generate random control for gene size-study validation

To validate the positive correlation between coding sequence size and rNMP incorporation frequency per base (PPB) on the non-template strand, we generated three random rNMP libraries as control. A customized python3 script is used to generate these three rNMP libraries with 10 000, 100 000 and 1 000 000 rNMPs randomly incorporated in hmtDNA. The PPB values in these random libraries are calculated and compared with real rNMP libraries.

### Mass spectrometry analysis of nucleotides

dNTP and rNTP concentration (pmol/million cells) for activated CD4^+^T cells were collected from a previous study having 4 replicates, and the precalculated average was used to represent dNTP/rNTP ratios (41). The nucleotide samples were extracted based on the established protocol (42) with some modifications. To prepare the nucleotide samples, 2 × 10^6^ cells of HEK293T or hESC-h9 were counted and centrifugated to obtain a cell pellet. The pellet was washed with PBS and then vortexing was performed for 2 min with 200 μl of cold 65% methanol for cell lysis. The cell mixture was incubated at 95°C for 3 min and then incubated on ice for 1 min to complete the cell lysis. By centrifugation at 14 000 rpm for 3 min, the supernatant containing nucleotides was isolated. To quantify the intracellular dNTPs and rNTPs, an ion pair chromatography-tandem mass spectrometry method (43) was applied, with modifications. Chromatographic separation and detection were performed on a Vanquish Flex system (Thermo Fisher Scientific) coupled with a TSQ Quantiva triple quadrupole mass spectrometer (Thermo Fisher Scientific). Analytes were separated using a Kinetex EVO-C18 column (100 × 2.1 mm, 2.6 μm) (Phenomenex) at a flow rate of 250 μl/min. Pmol/million cells were calculated for rNTPs and dNTPs for all four replicates to calculate the ratios.

### Human genes in the study

The human gene annotation GTF file was downloaded from the human reference genome GRCh38 in Ensembl genome browser 108. The mtDNA genes were selected from the GTF file. CDS and non-coding genes were also indicated in the same file. The processed gene locations are available on Zenodo (https://doi.org/10.5281/zenodo.10211459).

### Compare light and heavy strand sequencing bias using DNA sequencing

Whole genome sequencing data of genomic DNA samples extracted for ribose-seq were also obtained using the Illumina NextSeq 500 System with Mid-output (PE150) in the Molecular Evolution Core Facility at the Georgia Institute of Technology. The FASTQ reads were trimmed to remove any residual Illumina adapters and filtered to keep reads above the quality threshold of 15 and length 50 bp. These trimmed reads were then aligned using Bowtie 2 (44) with the hg38 reference genome. The reads aligned to the light and heavy strands of mtDNA were calculated to check any strand-biased sequencing. The coverage-mean depth was calculated per base pair using SAMtools and was greater than 30x for all libraries.

### Strand bias contribution of each type of rNMPs

To calculate the contribution of each type of rNMP to the strand bias distribution of rNMPs in hmtDNA, the count of each type of rNMP is divided by the background dNMP count to get the rNMP embedment rate for the light and the heavy strand, respectively. Then, for each type of rNMP, the strand-incorporation difference is calculated as the difference in rNMP incorporation rate between the preferred rNMP embedment strand and the other strand in each library. The positive difference suggests that a type of rNMP contributes to the excess of the preferred rNMP-embedment strand in the library. Afterward, the contribution percentage is calculated using all the positive differences. To make a further normalization on the strand bias level, the contribution percentage is multiplied by the difference of the rNMP embedment percentage to obtain the final contribution. As a result, this contribution is normalized on the background genome, total rNMP counts and strand bias level. Thus, it is comparable among all types of rNMPs in different libraries.

### Statistical test

To test if a dinucleotide or trinucleotide pattern was preferred in human or yeast mtDNA, a one-tailed Mann–Whitney *U* test was used to compare the normalized frequency for each dinucleotide or trinucleotide pattern and the expectation value (0.25 for dinucleotide and 0.0625 for trinucleotide). To test if one dinucleotide or trinucleotide pattern was preferred on the light or the heavy strand, a two-tailed Mann–Whitney *U* test was performed. For both statistical tests, the significance level of 0.05 was used.

### Visualizations

Alignment of ribose-seq data and finding the coordinates was done using Ribose-Map (30,45). The rNMP composition and dinucleotide heatmaps were generated using RibosePreferenceAnalysis (33). All other figures were generated using Python3 scripts, which are available on Zenodo (https://doi.org/10.5281/zenodo.10211459). Detection of common and high-frequency rNMP locations with ggseqlogo plot visualizations was conducted using R scripts available on Zenodo (https://doi.org/10.5281/zenodo.8152071). Figure 2 is generated with the help of SnapGene® software (from Dotmatics; available at snapgene.com).

## Results

### rNMP-embedment preference on the light strand of hmtDNA

**Figure 1.**
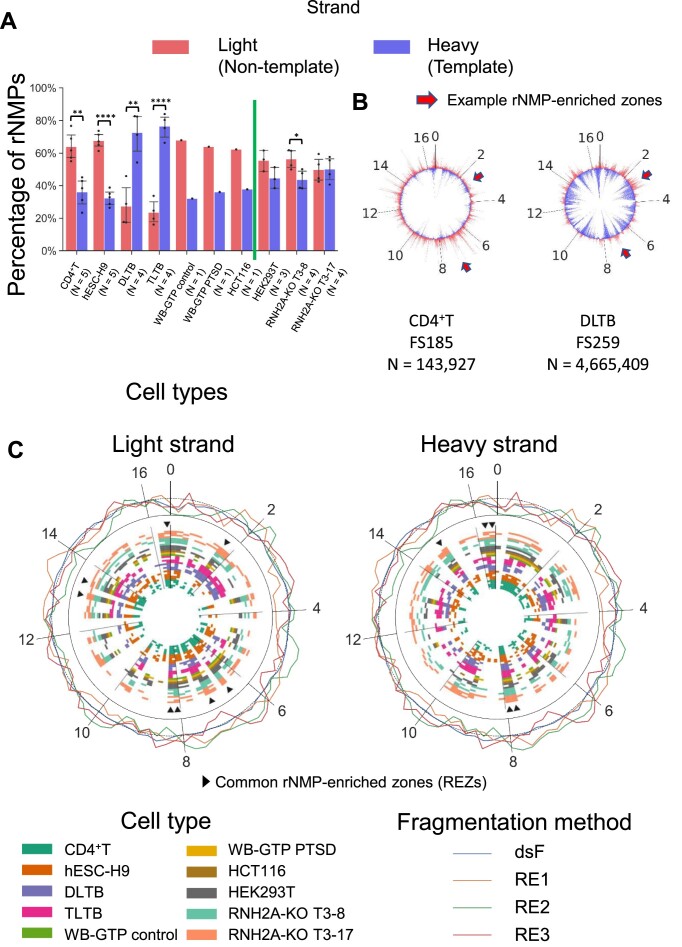
Biased rNMP distribution in hmtDNA. (**A**) Bar graph showing the percentage of rNMP embedment on the light (red bars) and heavy (blue bars) strands in hmtDNA. The cell type is indicated below each bar. The green vertical line separates cell types with RNH2A WT and KO. Two-tailed Mann–Whitney *U* test is performed to check the significance of the light/heavy strand bias for all cell types with *N* ≥ 3. *: 0.01 < *P* < 0.05; **: 0.001 < *P* < 0.01; ***: 0.0001 < *P* < 0.001; ****: 0.00001 < *P* < 0.0001. (**B**) rNMP frequency at each nucleotide on the light (outer-circle red spikes) and heavy (inner-circle blue spikes) strands of hmtDNA. The maximum spike heights represent the half of maximum rNMP count on the light and heavy strands indicated in Supplementary Table S7. Examples of consistent enrichment of rNMPs on the light strand are indicated by red arrows. (**C**) The rNMP-enriched zones (REZs) on the light and heavy strands of hmtDNA. Each circle represents one library. Each block in each circle represents a REZ, which is a 200-nt zone having an rNMP-enrichment factor > 1. The color of the circles represents the cell type and genotype. The common REZs are 200-nt zones that are REZs in all cell categories and genotypes, and more than 80% of the libraries studied. The common REZs are indicated by the triangle markers and listed in Supplementary Table S3. The outer circle shows the background coverage of RE and dsDNA Fragmentase. The mean background coverage of the whole hmtDNA (enrichment factor = 1) is represented as the dotted line in the outer circle.

The two strands of hmtDNA are named the light strand and the heavy strand, due to their differences in molecular weight and GC skew, with the heavy strand being richer in dGMPs than dCMPs. Twelve of the 13 CDSs are located on the light strand and are transcribed as one polycistronic mRNA of the light strand from the heavy-strand promoter (HSP) using the heavy strand as a template. One CDS, ND6, is transcribed in the opposite direction as another polycistronic mRNA with some non-coding genes from the light-strand promoter (LSP) (46). In this study, we constructed 32 ribose-seq libraries from mtDNA of six different human cell types: CD4^+^T lymphocytes, embryonic stem cells (hESC-H9), cells derived from distal or tumor liver-tissue biopsies (DLTB and TLTB), whole blood cells from a patient affected by post-traumatic stress disorder (PTSD) and control (WB-GTP PTSD and WB-GTP control), colorectal carcinoma cell line (HCT116), and human embryonic kidney cells (HEK293T), having either wild-type ribonuclease (RNase) H2, or having the catalytic subunit of RNase H2 knocked-out (RNH2A-KO) in two different cell clones (RNH2A-KO T3-8 and RNH2A-KO T3-17) (Supplementary Table S2, see Material and Methods). These cells are classified in our study into 10 different cell categories: CD4^+^T, hESC-H9, DLTB, TLTB, WB-GTP control, WB-GTP PTSD, HCT116, HEK293T, RNH2A-KO T3-8 and RNH2A-KO T3-17. In the preparation of the ribose-seq libraries, we utilized different fragmentation methods to cut hmtDNA, including dsDNA fragmentase, three distinct restriction enzyme (RE) combinations optimized using the RESCOT software (31), and dsDNA fragmentase together with RE combinations. With the paired-end sequencing reads, we adapted the Ribose-Map protocol (30,40) to align the rNMPs to the circular mtDNA of *Homo sapiens* GRCh38 reference genome and recovered the unaligned rNMPs at the beginning and end in the reference sequence (See Material and Methods). The library information including cell types, RNase H2 genotypes, fragmentation methods, and count of rNMPs is presented in Supplementary Table S2. We found that the rNMP embedment in hmtDNA of the six cell types studied is biased between the light and the heavy strands. Specifically, rNMPs are preferentially embedded on the light strand, which is the non-template strand relative to transcription of the long transcript generated from the HSP, in most of the ribose-seq libraries of the cell types examined. In the libraries derived from HEK293T cells that are wild-type or knocked out for RNase H2A, the bias is less prominent or not significant. Interestingly, the ribose-seq libraries derived from liver-tissue samples (DLTB and TLTB) show a dominant presence of rNMPs on the heavy strand, or template strand of hmtDNA (Figure 1A).

As a comparison, we performed the same strand-distribution analysis of embedded rNMPs in budding yeast mtDNA. We analyzed 11 ribose-seq libraries derived from wild-type RNase H2 strains of *S. cerevisiae* and 8 libraries derived from mutant-RNase H2 strains with the catalytic subunit deleted (*rnh201-*null) that were built in a previous study (21). These yeast libraries derive from six different strains and have been fragmented using three different RE combinations. The *S. cerevisiae* mtDNA contains 35 genes, including 8 CDS, 1 ribosomal protein, 2 rRNA genes and 24 tRNA genes (47). All yeast mtDNA CDSs are located on the forward strand. By analyzing these yeast libraries, we found that the rNMPs are preferentially embedded on the reverse strand in both wild-type and *rnh201*-null libraries (Supplementary Figure S1A). Considering that the reverse strand is the template strand of all CDSs in yeast mtDNA during the process of transcription, yeast mtDNA has an opposite strand bias of rNMP distribution to that found in hmtDNA relative to transcription in most of the human cell types studied here, but a similar bias to that found in hmtDNA libraries derived from the liver-tissue cells.

### rNMP-enriched zones and rNMP hotspots in hmtDNA

To better understand the rNMP-embedment characteristics in hmtDNA, we plotted the rNMP distribution maps at the single-nucleotide level (Figure 1B and Supplementary Figure S2). From these maps, we found that the rNMPs are distributed unevenly along the strands of the hmtDNA. We identified some genomic zones with enrichment of incorporated rNMPs, which have higher rNMP-embedment frequency relative to the hmtDNA average. We termed these zones as rNMP-enriched zones (REZs). We calculated the rNMP-enrichment factor of all 166 bins of 200-nt size covering both mtDNA strands for all the rNMP libraries within the 10 cell categories listed above and we identified the REZs. Surprisingly, we found that the distribution of REZs in the different libraries shows high consistency (Figure 1C). We revealed eight common REZs on the light strand and five common REZs on the heavy strand, which are present in all 10 cell categories and more than 80% of the libraries (Figure 1C, Supplementary Table S4). The presence of these common REZs is therefore independent of the cell type. To check if the REZs represent an artificial result caused by the different methods of hmtDNA fragmentation, we performed DNA-seq and calculated the read coverage in CD4^+^T libraries fragmented by each of the three RE combinations, and by dsDNA Fragmentase (outer circle in Figure 1C). Results show that the libraries constructed using dsDNA Fragmentase keep a consistent coverage all over the mtDNA. The libraries prepared using restriction enzymes (REs) have some fluctuation among different hmtDNA regions due to the different recognition patterns. However, the common REZs are not enriched in either high-read-coverage regions or low-read-coverage regions, suggesting that the common REZs are uncorrelated with the different fragmentation methods. Hence, the common REZs are intrinsic properties of rNMP embedment in hmtDNA.

To determine whether REZs are present in the yeast mtDNA, we generated the rNMP-distribution maps of previously published ribose-seq and emRiboSeq libraries of *S. cerevisiae* (21,25). Similarly to what was found in hmtDMA, among all the yeast mtDNA ribose-seq libraries analyzed, common REZs existed independently from (i) the RNase H2 genotype; (ii) the RE set used for fragmenting the genome (RE1, RE2 or RE3); (iii) the rNMP-mapping technique used, which is particularly relevant because emRiboSeq utilizes a protocol for genome fragmentation and capture of rNMPs different from ribose-seq (25) (Supplementary Figure S1B). Overall, the results obtained for human and yeast mtDNA show an uneven distribution of rNMPs in the mtDNA genome and highlight the presence of preferred regions of rNMP embedment. The consistency of common REZs across the human and yeast mtDNAs that we studied may reflect specific metabolic activities or structural features of mtDNA.

**Figure 2.**
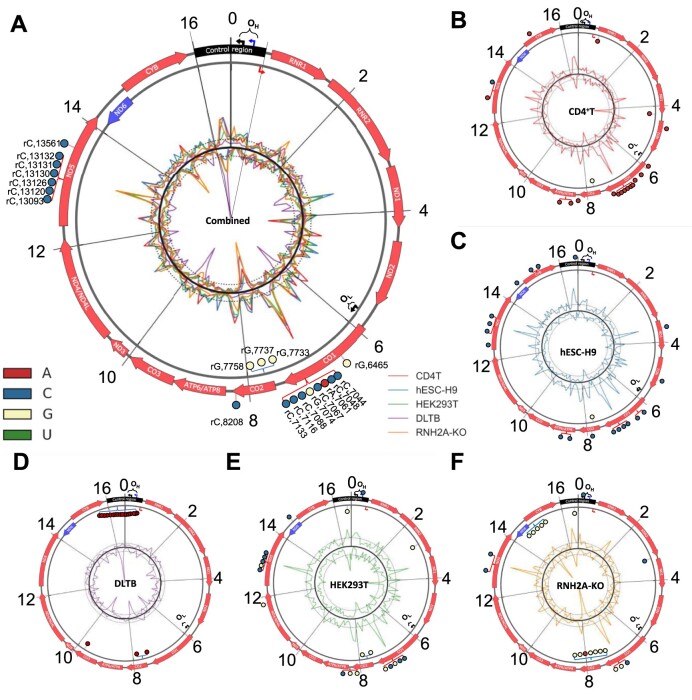
*rNMP hotspots in hmtDNA*. Circular plots showing the rNMP-embedment frequency and rNMP hotspots in hmtDNA for (**A**) all hmtDNA libraries, (**B**) CD4^+^T, (**C**) hESC-H9, (**D**) DLTB, (**E**) HEK293T RNH2A WT and (**F**) HEK293T RNH2A KO libraries (Both RNH2A-KO T3-8 and RNH2A-KO T3-17). The coding sequences on the light and heavy strands are indicated as red and blue arrows on the outer circle. The control region is marked as black on the top. Two black braces represent the replication origin regions of the heavy strand (OriH, OH) and light strand (OriL, OL), and the black bent arrows show the actual replication start sites. The blue and red bent arrows indicate the heavy-strand promoter (HSP) and light-strand promoter (LSP), respectively. The outer and inner lines of the inner circle are the rNMP-enrichment factor of the light and heavy strands of cell categories containing more than 3 libraries. Two dotted lines around the circle indicate the mean value of rNMP-embedment frequency (Enrichment factor = 1. See Materials and methods). The top 20 common hotspots in each subset and all hmtDNA on the light and heavy strands are marked as colored pins in the outer circle. The types of rNMP hotspots are shown in different colors. The type and location of all hotspots are also listed in Supplementary Table S4.

The rNMP hotspots are single-nucleotide locations with one or more rNMPs in all of the 32 libraries studied. We calculated the average rNMP-embedment enrichment factor for libraries derived from the same cell type and found that the rNMP hotspots are present in regions of high rNMP-embedment frequency (Figure 2). We identified common rNMP hotspots in hmtDNA in all libraries we analyzed (Figure 2A), as well as unique rNMP hotspots for CD4^+^T, hESC-H9, DLTB, HEK293T and HEK293T RNH2A-KO (RNH2A-KO T3-8 and RNH2A-KO T3-17) subsets (Figure 2B–F, Supplementary Table S5). The top 20 hotspots with the highest median enrichment factor are selected and marked in Figure 2. Most selected rNMP hotspots (17 out of 20) in the combined-hmtDNA libraries are present on the light strand and inside the non-template strand of the coding sequences (Figure 2A). Most of them are clustered together in two windows of ∼100-nt length located on the non-template strand of the NADH dehydrogenase 5 (MT-ND5) gene, and the Cytochrome C Oxidase subunit I (MT-CO1) gene. 70% of the common-rNMP hotspots are rCMPs, suggesting that rCMPs are strongly preferred as the rNMP hotspots in hmtDNA. Considering the individual subsets (CD4^+^T, DLTB, hESC-H9, HEK293T and HEK293T RNH2A KO), we identified abundant rNMP hotspots and selected the top 20 (Figure 2B–F, Supplementary Table S5). The rNMP hotspots in each subset have different strand preferences that are in line with the strand-biased distribution of the rNMPs in the subset. For CD4^+^T, hESC-H9 and HEK293T libraries, there are more rNMP hotspots on the light strand (Figure 2B, C, E). On the contrary, in the DLTB libraries, we see rNMP hotspots mainly present on the heavy strand (Figure 2D), which is consistent with the dominant presence of rNMPs on the heavy strand in these libraries (Figure 1A). The clusters of rNMP hotspots exist in the control region in DLTB libraries and on the template strand of Cytochrome B (MT-CYB) and Cytochrome C Oxidase II (MT-CO2) genes in the HEK293T RNH2A-KO cells (Figure 2D, F). Moreover, the rNMP hotspots in each subset show a strong composition preference. In the CD4^+^T and DLTB libraries, almost all rNMP hotspots are rAMPs (CD4^+^T: 19 out of 20, Figure 2B; DLTB: 20 out of 20, Figure 2D). In the hESC-H9 libraries, almost all rNMP hotspots are rCMPs (19 out of 20, Figure 2C). In the HEK293T libraries, all rNMP hotspots are rCMPs or rGMPs (Figure 2E), and in the HEK293T RNH2A-KO libraries, the most rNMP hotspots are rGMPs (14 out of 20, Figure 2F). The consistency of the rNMP hotspots may play a vital role in hmtDNA gene function, especially in the clustering regions.

### rNMP-embedment characteristics in the control region of hmtDNA

**Figure 3.**
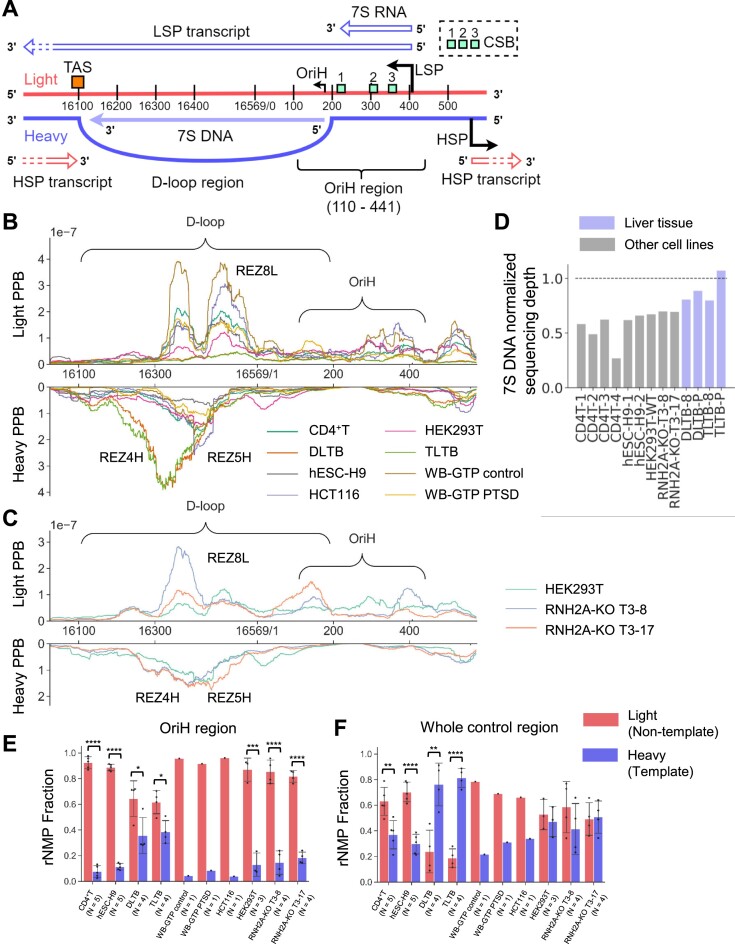
Conserved rNMP-enriched zones in the control region. (**A**) Scheme of genetic elements in the hmtDNA control region. (B, C) rNMP-embedment probability per base (PPB) in the hmtDNA-control region for (**B**) all RNH2A WT-cell types and (**C**) all HEK293T libraries with RNH2A KO or WT. The rNMP PPB on the light strand is on the upper part, while the rNMP PPB on the heavy strand is on the bottom part. The common REZs in the control region are listed in the figures. Detailed information regarding these REZs is available in Supplementary Table S3. (**D**) The normalized sequencing depth of 7S DNA and its complementary sequence in all cell types. The dashed line shows the mean sequencing depth of whole hmtDNA in each library. (E, F) The rNMP percentage on the light (red) and heavy (blue) strand for (**E**) OriH region and (**F**) whole control region in mtDNA. Two-tailed Mann–Whitney *U* test is performed to check the significance of the leading/lagging strand bias for all cell types with *N* ≥ 3. *: 0.01 < *P* < 0.05; **: 0.001 < *P* < 0.01; ***: 0.0001 < *P* < 0.001; ****: 0.00001 < *P* < 0.0001.

There is only one long non-coding region in the hmtDNA located around the zero position in the hmtDNA (black box in Figure 2), which is called the control region and contains essential genetic elements for mtDNA transcription and replication including HSP, LSP, and the termination-associated sequences (TAS) (Figure 3A) (48). The heavy-strand replication origin (OriH, OH) and the D-loop, a special three-strand region containing a displaced light strand, a heavy strand, and a hmtDNA replication failure-product 7S DNA are also present in the control region. We identified three rNMP peaks in the control region, two on the light strand, one of which is REZ8L, and one on the heavy strand, which corresponds to REZ4H and REZ5H, located in the D-loop region right before the OriH (Figure 3B, C and Supplementary Table S3). These three peaks exist in all subsets and cell types we analyzed (Supplementary Figure S3). The abundance of embedded rNMPs in these peaks is in line with the strand preference of the whole hmtDNA in the different cell types. For most cell types, there are more embedded rNMPs in the light-strand peaks. However, the liver-tissue libraries, DLTB and TLTB, have fewer rNMPs in the light-strand peak (REZ8L) and more rNMPs in the heavy-strand peak (REZ4H and REZ5H) (Figure 3B). Considering that the 7S DNA has the same DNA sequence as the D-loop heavy strand, the copy number of the 7S DNA may be related to the strong heavy-strand peak in liver tissue mtDNA. Hence, we measured the abundance of 7S DNA normalizing to the sequencing depth. Indeed, we found that the liver tissue libraries have higher abundance of the 7S-DNA and its complementary sequence (Figure 3D), as expected after sequencing if the hmtDNA of the liver tissue cells would more often contain the three-strand D-loop structure than the other cell types. Abundant rNMP hotspots are also clustered in the same zone on the heavy strand in DLTB libraries (Figure 2D). Compared to the wild-type RNase H2 cells, similar and stronger peaks exist in HEK293T RNH2A-KO libraries (Figure 3C and Supplementary Figure S3). Moreover, we compared the rNMP-embedment frequency in the control region to the published ChIP-seq data of DNA Pol γ and mtDNA helicase TWINKLE (49). We found that the ChIP-seq signal is lower in the rNMP peaks compared to the surrounding regions, which suggests that Pol γ and TWINKLE protein occupancy is lower in the rNMP peaks of both strands (Supplementary Figure S4).

In hmtDNA, the light-strand transcripts start at LSP. The same LSP promoter produces an abundant short non-coding RNA, 7S RNA, which is not bound to the DNA template (50). Moreover, some LSP transcripts are prematurely terminated, serve as primers for heavy-strand replication, and form persistent RNA/DNA hybrids (50–52). The transition from terminated, priming RNAs to DNA happens at many different points around two conserved sequence blocks (CSB), CSB2 and CSB3 (53–56). Hence, the region containing these transition points has been known as the OriH region (1,53) (Figure 3A). In this region, there are significantly more rNTPs incorporated on the light strand in all cell types (Figure 3E). Even in the liver-tissue cells and HEK293T RNH2A-KO T3-17 libraries, where rNTPs are preferentially incorporated on the heavy strand in the whole hmtDNA, there are more rNTPs incorporated on the light strand in the OriH region. In contrast, the strong light-strand preference does not exist in the whole control region (Figure 3F).

### Longer CDSs have a higher rNMP-embedment frequency on the non-template, light strand

**Figure 4.**
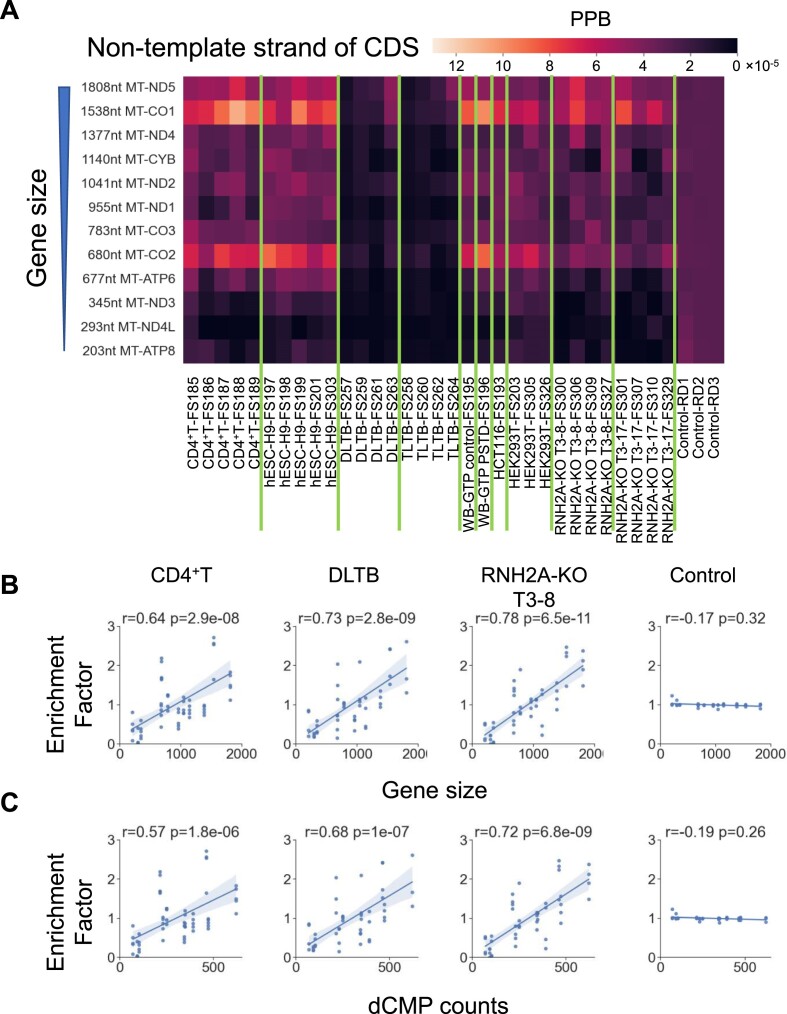
Longer CDSs have a higher rNMP-embedment frequency on the non-template strand, the light strand. (**A**) rNMP-embedment probability per base (PPB) on the non-template strand of each coding sequence located on the light strand in each library. The PPB measures the normalized rNMP-embedment frequency at each base (see Materials and methods). Each row represents a CDS, which is sorted by descending gene size. Each column represents an rNMP library. There are three control libraries with artificial rNMP embedment generated *in silico* on the right (see Materials and methods). (B, C) Linear-regression plot showing a positive relationship between the rNMP-enrichment factor and the (**B**) gene size and (**C**) dCMP count on the non-template strand. Each data point represents the rNMP-enrichment factor on the non-template strand of a particular gene in an rNMP library of a given cell type. The rNMP-enrichment factors are calculated as described in Materials and methods. Spearman's *r* and *P*-values are marked in the plot. Cell types with Spearman *r* > 0.5 have a strong positive correlation between the rNMP-enrichment factor and the gene size. The 95% confidential interval is marked as the blue shadow region around the line.

We checked the rNMP-embedment frequency on both strands of the 12 CDSs located on the light strand of the hmtDNA. The rNMP-embedment frequency in the CDS region is in line with the strand preference of the whole mtDNA. In most cell types, there are abundant rNTPs incorporated on the light strand of DNA, which is the non-template strand of the sense transcript for these 12 CDSs. In the liver-tissue libraries, there are more rNTPs incorporated on the heavy strand, which is the template strand of the sense transcript for these CDSs. An unexpected finding is that the longer CDSs have more rNTPs incorporated on the non-template strand (light strand), even after the normalization by CDS size (Figure 4A). A significant positive correlation between CDS size and rNMP-embedment frequency is detected in all cell types despite the strand preference and RNase H2 genotype (Spearman's *r* > 0.6, Figure 4B). Of note, the higher rNMP-embedment rate on the non-template, light strand of the longer vs. shorter CDSs correlates with the dCMP content of the genes on the non-template, light strand (or with the dGMP content on the template, heavy strand) (Figure 4C). To validate our finding, we generated three random libraries with 10 000, 100 000 and 1 000 000 rNMPs incorporated in DNA as the control and calculated their PPB (see Materials and methods). The results show that the CDS size is irrelevant to the rNMP-embedment frequency in the control group on the non-template strand (Spearman's *r* < 0.2, Figure 4A). There is no correlation between the CDS size and rNMP embedment on the template strand (Supplementary Figure 5A–C), or both template and non-template strands in yeast mtDNA (Supplementary Figure S6A, B).

There are 2 rRNA and 22 tRNA genes in the hmtDNA. The longer rDNA (MT-RNR2) has a higher rNMP-embedment frequency than the shorter one (MT-RNR1) in all libraries on the non-template strand. The tRNA genes have similar lengths ranging from 58 to 74 nt. Given the short length of tRNA genes, few rNMPs are incorporated, which leads to high variation in the frequency and non-significant association between tRNA gene length and rNMP embedment frequency (Supplementary Figure S5D).

### The composition of incorporated rNMPs in hmtDNA of different cell types

**Figure 5.**
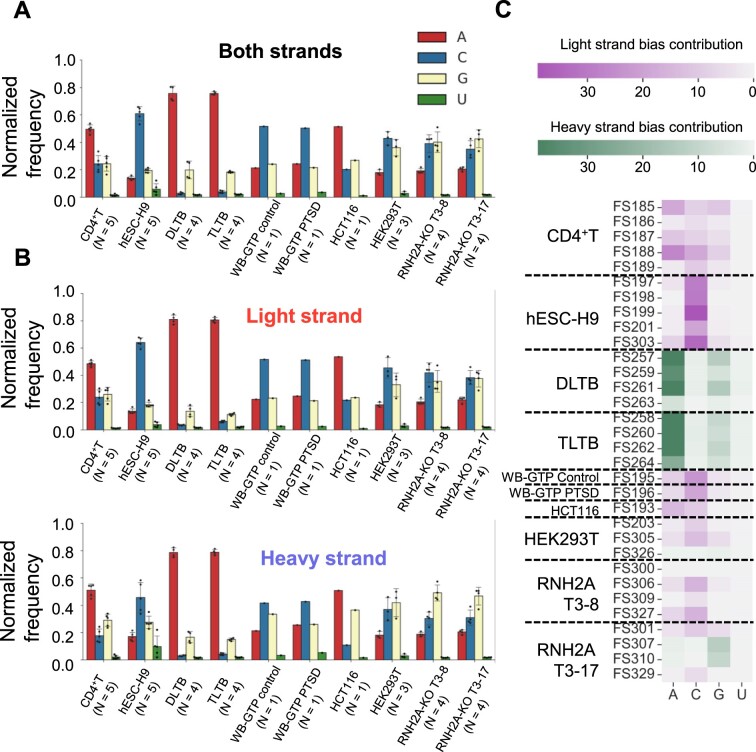
*The rNMP composition on both strands of hmtDNA*. (A–C) Bar plot showing the normalized frequency of the four rNMP types on (**A**) both strands, (**B**) light strand, and heavy strand of hmtDNA. The normalized frequency is calculated as described in the Materials and methods section. Types of rNMPs and their corresponding colors are listed in the color legend on the right top. (**C**) Heatmap showing the contribution of each type of rNMPs to the strand bias (see Materials and methods). The color intensity measures the relative contribution to the rNMP-embedment preferred strand (purple for the light strand and green for the heavy strand).

The rNMP-embedment analysis of the 32 hmtDNA libraries reveals that rUMPs are the least abundant rNMPs in all cell types despite the strand preference and RNase H2 genotype (Figure 5A). Interestingly, rCMP is found quite low only in the DLTB and TLTB cells. The preferred rNMPs vary among the cell types. In CD4^+^T, liver-tissue (DLTB and TLTB) and HCT116 libraries, rAMPs are preferred. In hESC-H9, WB-GTP control, WB-GTP PTSD and HEK293T RNH2A WT libraries, rCMPs are preferred. In the HEK293T RNH2A-KO libraries, both rCMPs and rGMPs are preferred (Figure 5A and Supplementary Figure S7A). We located the high frequency-rNMP sites (top 1% sites having the most abundant embedded rNMPs, see Materials and methods) in mtDNA and analyzed their sequences. These high frequency-rNMP sites also show similar preferred rNMPs as in the whole hmtDNA (Supplementary Figure S8, 0 position is the rNMP position). CD4^+^T, liver tissue (DLTB and TLTB), and HCT116 cells show preferences for rAMPs in most libraries, whereas hESC-H9 libraries have a strong preference for rCMPs. HEK293 RNH2A WT and KO cells show preferences for rCMPs and rGMPs.

Our analysis reveals a difference in rNMP composition between the light and heavy strands of hmtDNA. The light strand has more rCMPs incorporated than the heavy strand in all cell types, (Figure 5B and Supplementary Figure S7B). Furthermore, we analyzed the strand bias contribution of each type of rNMPs to the excess of rNMPs on the preferred strand. The result shows that the excessive rNMP embedment on the light-strand is predominately induced by rCMPs (Figure 5C). Differently, the excess of rNMPs found on the heavy strand compared to the light strand of hmtDNA in DLTB and TLTB cells (Figure 1A) is due to the abundant presence of rAMPs (Figure 5C). In yeast mtDNA, the reverse strand has slightly more rCMPs than the forward strand (Supplementary Figure S9). Moreover, the rNMP compositions do not match the rNTP abundancy relative to that of the dNTPs in the cells (Supplementary Figure S10). These results suggest that the rNTP and dNTP pools are not the only determinants for rNMP-embedment composition and patterns, and other cellular functions may differentially condition the rNMP presence on the light and heavy strands of hmtDNA.

### Sequence context of the embedded rNMPs

**Figure 6.**
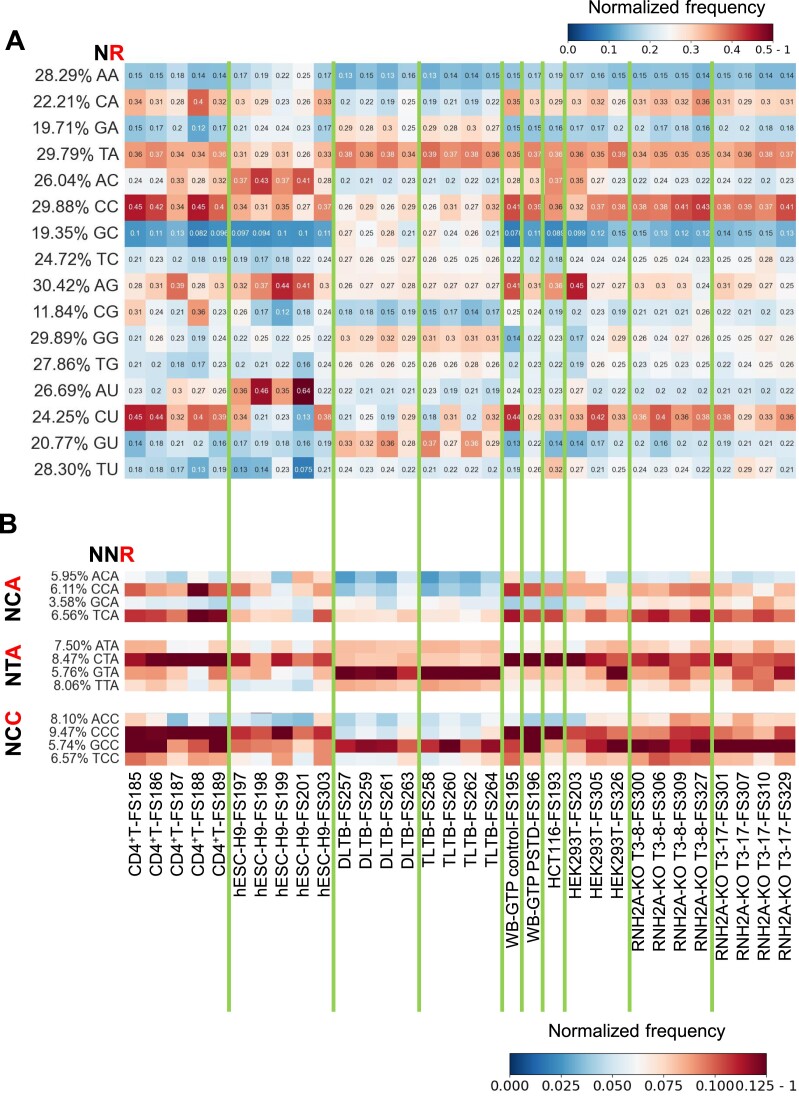
Preferred rNMP-embedment patterns in hmtDNA. (**A**) Heatmap analyses with the normalized frequency of dinucleotides composed of the incorporated rNMP (R: rA, rC, rG, or rU) and its upstream dNMP neighbor (N: dA, dC, dG, or dT) (NR) in the hmtDNA. The rNMP position in the dinucleotide is shown in red at the top left of the heatmap. Each column of the heatmap shows the results of an rNMP library. The vertical, green lines separate different cell types. The color scale is shown in the top right corner of the figure. (**B**) Heatmap analyses with the normalized frequency of selected trinucleotides composed of the incorporated rNMP (R: rA, rC, rG, or rU) and its two upstream dNMP neighbors (N: dA, dC, dG, or dT) (NNR) in the hmtDNA. The color scale is shown in the bottom right corner of the figure.

Distinct dinucleotide patterns consisting of the incorporated rNMP and its direct upstream dNMP neighbor are revealed in this study. Specifically, TrA, CrC and ArG are preferred in all cell types while CrA and CrU are preferred in all cell types except liver-tissue cells (Figure 6A, *P* < 0.05 in Supplementary Table S5). Compared to other cell types, liver-tissue cells have more dGMPs located upstream of all four types of rNTPs (Figure 6A).

Similar to what was observed in yeast mitochondrial and nuclear DNA (21), there are fewer dinucleotide patterns formed by rNMPs and their downstream dNMP (RN) due to the downstream dNMP being incorporated following the incorporation of the rNMP. The only two RN patterns we identified were rAG and rUA in liver-tissue samples. There is no strong RN pattern in other cell types (Supplementary Figure S11).

Our analysis reveals that there is a strand bias for particular dinucleotide patterns (NR). We found that CrU is stronger on the light strand in the HEK293T RNH2A-KO libraries while CrC is stronger on the heavy strand in the liver-tissue and HEK293T RNH2A-KO libraries (Supplementary Figure S12, *P* < 0.05 in Supplementary Table S6). In contrast, the forward and reverse strands of mtDNA have quite similar dinucleotide patterns in yeast. CrA, ArC, ArG, and CrU are consistently preferred in both strands of yeast RNase H2 WT and *rnh201*-null libraries ((21) and Supplementary Figure S13).

Furthermore, we identified preferred trinucleotide patterns in hmtDNA. GTrA and GCrC are strong in all cell types but preferred in liver-tissue libraries. CCrA, TCrA, CTrA, and CCrC are preferred in all libraries except liver-tissue libraries. (Figure 6B and Supplementary Figure S14A, *P* < 0.05 in Supplementary Table S4). The light and heavy strands have many different preferred trinucleotide patterns. For example, there are stronger CCrA and CCrG patterns on the light strand and more AArG and ATrG on the heavy strand. (Supplementary Figure S14). In the yeast libraries, there are biased trinucleotide frequencies, including stronger GTrC on the forward strand and stronger CCrA on the reverse strand (Supplementary Figure S15).

## Discussion

Previous analyses that mapped rNMPs embedded in hmtDNA revealed a significant correlation between nucleotide pools, the identity, and frequency of the rNMPs in hmtDNA when nucleotide pools were altered. However, these analyses offered limited insights into the characteristics of rNMP embedment and their conservation across different cell types (12). To gain a broader understanding of the features of rNMP embedment in hmtDNA across various human cell types, along with its potential sources and functions, we conducted a thorough and detailed analysis of rNMPs in hmtDNA. Through the analysis of 32 libraries of rNMP sites embedded in mtDNA of six different human cell types, the results of this study support non-random rNMP presence in hmtDNA starting from an rNMP-embedment bias on the light strand of hmtDNA of most cell types examined except for the liver-tissue samples (Figure 1A). The work by Berglund *et al.* (12) highlights a higher level of rNMPs on the light strand in normal human fibroblasts but not major strand bias in HeLa cells. We found that different cell lines showed a disparity in rNMP embedment strand bias, which might be specific to the different cell types and/or growth/environmental conditions of the samples analyzed, i.e. cell culture source from most DNA samples studied and tissue source for the liver DNA samples, and/or could potentially be related to the abundance of different replication intermediates of hmtDNA. Indeed, as discussed below, in the hmtDNA of the liver-tissue cells, the D-loop region formed during the synthesis of the new heavy strand appears to be more abundant than in the other cell types.

**Figure 7.**
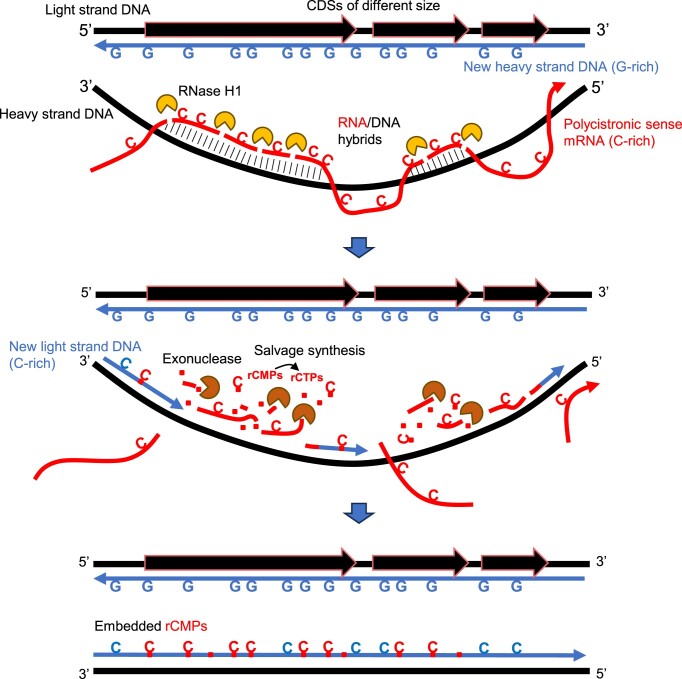
Model for enriched rCMP embedment on the non-template, light strand of the large hmtDNA CDSs and its proposed function. The diagrams show the proposed mechanism of rCMP overload on the non-template, light strand with enrichment in the large coding sequences (CDSs). RNA-DNA hybrids are more likely to form between the displaced heavy-strand DNA and the sense-polycistronic mRNA in the C-rich regions corresponding to the large CDSs. Before DNA replication of the light strand starts, RNase H1 resolves the hybrids into short RNA fragments, which are further degraded into rNMPs by other mitochondrial nucleases. The rCMPs can be converted into rCTPs through nucleotide-salvage synthesis directly in mitochondria, leading to higher rNTP abundance around the heavy, template strand of the large CDSs. The locally abundant rCTPs may be frequently incorporated by DNA Pol γ during new light-strand synthesis.

Unexpectedly, we found a significant positive correlation between CDS length, length of tracts with GC skew, and rNMP-embedment frequency on the non-template (light) strand (Figure 4). We believe that this correlation is related to the asymmetric DNA replication mechanism of hmtDNA and the formation of extensive tracts of RNA-DNA hybrids between the polycistronic sense-transcript RNA and the DNA-heavy strand (53,54). The abundant GC skew of the hmtDNA strands and the fact that long sense and antisense RNA molecules are transcribed along the same region in hmtDNA might favor the formation of R-loops i.e. RNA–DNA hybrids (57). The CDSs have the same level of transcription, being transcribed in the same long polycistronic RNA. Eliminating the transcription variable, the probability of RNA–DNA hybrid formation across large versus small CDSs might be strongly determined by the excess of C versus G in the CDSs. Thus, large CDSs could have a higher probability of RNA–DNA formation hybrids than small CDSs. The ribonucleotide incorporation throughout the lagging strand (RITOLS) model (42,58–60) is an asynchronous model of hmtDNA light-strand replication, which involves the formation and successive removal of RNA–DNA hybrids. These hybrids are established between the sense mRNA and the heavy-strand DNA template. During this process, different parts of the lagging strand are incorporated as RNA before the synthesis of DNA commences. In line with the RITOLS model, and because rNMPs are mainly found in DNA due to the incorporation of rNTPs by DNA polymerases (3,4), and the ribose-seq procedure only captures rNMPs that are fully incorporated in DNA, not RNA primers of Okazaki fragments (22), we suggest that a significant fraction of the incorporated rNTPs during light-strand synthesis may be enriched with degradation products of the sense, light strand RNA, particularly rCMPs. RNA degradation likely by RNase H1 followed by other mitochondrial nucleases like REXO2 (37) could free rNMPs, including abundant rCMPs, since the light strand is C-rich. Afterward, rCMPs could be converted into rCTPs through nucleotide-salvage synthesis directly in mitochondria, as described in (38). Notably, *in vitro* experiments of DNA synthesis using human Pol γ in the presence of rNTPs have shown that rCTP is the most frequently incorporated nucleotide into the newly synthesized strand (12). In our study, the rCMPs contributed most to the rNMP overload on the light-strand vs. the heavy-strand in Figure 1A (Figure 5C). Differently, the rNMP overload on the heavy strand of the mtDNA of the liver-tissue samples is dominated by rAMPs (Figure 5C). It is theoretically possible that Pol γ could exhibit strand-bias incorporation of rNTPs; however, there is currently no evidence supporting such a phenomenon. Therefore, we propose that the source of rCMP-bias on the non-template, light strand originates from the degradation of the sense transcript hybridized to the DNA-heavy strand. Such locally abundant rCMPs converted into rCTPs might be frequently incorporated by DNA Pol γ during light-strand synthesis, considering the asynchronous synthesis of the hmtDNA strands (Figure 7). On the other hand, the main source of the rAMP overload found on the heavy strand of the liver tissue cells might be more dependent on the nucleotide pools, in which rATPs tend to be quite abundant together with rGTPs in most cell types analyzed (Supplementary Figure S10) and the rATP/dATP ratio is usually the largest obtained from nucleotides measurements in mammalian cells (61).

Additional evidence of non-random rNMP presence in hmtDNA is given by the identification of common zones with enriched rNMP presence in hmtDNA, which we term REZs (Figure 1C and Supplementary Table S3). Among all our mtDNA samples, we found eight common REZs on the light strand and five common REZs on the heavy strands (Figure 1C). These common REZs are present in the hmtDNA of all six cell types studied independently from the RNase H2 genotype. The REZs are not linked to the method of DNA fragmentation used. Although the different fragmentation methods lead to variant rNMP capture efficiency, all our libraries show rNMP enrichment in the same REZs. REZs also exist in yeast mtDNA (Supplementary Figure S1B). These results corroborate the non-random distribution of rNMPs in mtDNA and highlight the presence of DNA regions displaying rNMP accumulation. The REZs may be closely related to mtDNA structure and function.

In the D-loop of the control region in all cell types, we identified three rNMP peaks, which include REZ8L, REZ4H and REZ5H (Figure 3A–C and Supplementary Figure S3). Interestingly, in the same zones, mtDNA Pol γ and mtDNA helicase TWINKLE show lower protein occupancy compared to other regions in the D-loop (49) (Supplementary Figure S4). Considering that the incorporated rNMPs can alter the DNA structure and affect DNA-protein interaction (7,23), the low occupancy of Pol γ and TWINKLE may be induced by the enrichment of rNMPs. In hmtDNA, most of the replication-initiation events are prematurely terminated, generating the 7S-DNA product (48). Frequent rNMP embedment on both strands in the control region may be associated with this phenomenon in different ways. A study in a mouse model of Mpv17 deficiency suggested that the high level of rGMP found in the Mpv17-depleted mouse cells may be associated with mtDNA-replication failure (18). Thus, while it remains to be tested whether there is a connection between rNMP incorporation and the ChIP-seq data of Pol γ and TWINKLE, the peaks with the REZ8L on the light strand of the control region may contribute to DNA-replication failure events in hmtDNA generating the 7S DNA. On the other hand, the peaks on the heavy strand (corresponding to REZ4H and REZ5H) might be a consequence of the abundance of the 7S DNA. In fact, our results show that liver-tissue cells have a stronger heavy-strand peak in the control region compared to the other cell types (Figure 3A–C and Supplementary Figure S3), which we revealed to be associated with a higher abundance of the 7S DNA in liver-tissue cells compared to the other cell types (Figure 3D). Therefore, REZ4H represents an overestimation of the rNMP abundance in this heavy strand region, supporting the abundant presence of a D-loop structure in hmtDNA of the liver-tissue cells compared to the other cell types.

Beyond the REZs, we identified common rNMP hotspots in hmtDNA and specific rNMP hotspots for each cell type (Figure 2). Remarkably, these rNMP hotspots displayed a similar composition to the entire hmtDNA of the corresponding cell type. For instance, in CD4^+^T and DLTB libraries, we observed a preference for rAMP embedment in hmtDNA (Figure 5A). Intriguingly, nearly all rNMP hotspots in these subsets were rAMPs, with 19 out of 20 in CD4^+^T and all 20 in DLTB (Figure 2B and Figure 2D, respectively). This consistent composition between rNMP hotspots and the whole rNMP content in the hmtDNA was evident across all library subsets we examined (compare Figure 2 and Figure 5A). Hence, there is a clear association between rNMP composition in whole hmtDNA and hotspots. Furthermore, our findings indicate that these rNMP hotspots tend to be clustered in specific CDS regions of hmtDNA, including CO1 and ND5 (Figure 2A). We speculate that the genomic sequence of these CDS regions might also contribute to the formation of rNMP hotspots.

Embedded rNMPs may contribute to premature transcription termination in the OriH region. The three conserved sequence blocks (CSB1: 213–235, CSB2: 299–315, CSB3: 346–363) located in the human OriH region (nucleotides 110–441) are highly conserved sequences located in the control region of mtDNA in many vertebrate species, and frequent premature termination of light-strand transcripts have been observed in the CSBs (62,63). Markedly, we found strong light-strand rNMP-embedment preference in the OriH region of all cell types including the liver-tissue cells, in which 80% of rNTPs are incorporated on the heavy strand (Figure 3E). As shown in a recent study, the embedded rNMPs can impair RNA polymerase activity and lead to transcription termination (64). The high rNMP-embedment rate on the light strand may affect the POLRMT binding activity and contribute to premature transcription termination.

In hmtDNA, rUMP is the least abundant rNMP detected in all cell types studied after normalization to the nucleotide content of the hmtDNA genome and independently from the RNase H2 genotype of the cells. This is in common with the rNMP composition in mtDNA of other eukaryotic cells, such as budding (*S. cerevisiae* and *S. paradoxus*) and fission (*S. pombe*) yeast (21), and the unicellular green alga (*C. reinhardtii*) (34). As observed in yeast and algae, the rUTP/dTTP ratio calculated from the concentrations of all rNTPs and dNTPs in the pool of the human cells analyzed is the smallest, while the rATP/dATP and rGTP/dGTP are the largest ratios (Figure 5 and Supplementary Figure S10). However, in contrast to what was observed in *C. reinhardtii*, in which rAMP was by far the most abundant rNMP in the mtDNA and chloroplast DNA of the alga in line with a large rATP/dATP ratio (34), the rNTP/dNTP ratios of the bases A, C, and especially G, do not match the rNMP composition in the hmtDNA. The relatively lower rGTP incorporation rate compared to its abundance may in part be due to rGTP frequent interaction with proteins in human cells (65). Moreover, these inconsistencies between rNTP/dNTP ratios and rNMP composition in hmtDNA of the different cell types analyzed also suggest that human mtDNA Pol γ may have a base preference for rNTP incorporation in hmtDNA. In vitro human Pol γ has a preference for rCTP followed by rGTP (12). Analyses of rNMPs embedded in the mtDNA of HeLa cells revealed low rUMP as we find, but higher rGMP on both the heavy and light strands of the hmtDNA in the HeLa cells (12). While rUMP is consistently the least incorporated rNMP, also in accord with the *in vitro* results, the rNMP composition in the mtDNA of the HeLa cell does not match any of the compositions found in the hmtDNA of the cell types analyzed in this study. Moreover, we note that rCMP is almost as low abundant as rUMP only in the liver tissue samples (Figure 5). These results suggest that there is not a uniform rNMP-composition scheme in hmtDNA dictated by the base preference of rNTP incorporation by DNA Pol γ. Thus, while Pol γ-rNMP preference certainly has an influence, likely multiple factors can affect the rNMP composition in hmtDNA, and there is a major variation of rNMP composition in different cell types, possibly reflecting different DNA and/or RNA metabolic activities of the cells.

Previous studies in yeast and algae showed that the rNTPs are preferentially incorporated after a particular type of dNMP in DNA (dA, dC, dG and dT) (21,34,66). In hmtDNA, we identified several preferred dinucleotide and trinucleotide patterns. We found TrA and CrC to be the most conserved patterns in hmtDNA among all cell types tested (Figure 6A). These dinucleotides are preceded by dG (GTrA and GCrC) in the liver-tissue samples, while they are preceded by dC (CTrA and CCrC) in all other cell types (Figure 6B). These dG and dC biases are in line with the more abundant rNMP presence on the heavy strand (G-rich) for the liver-tissue samples as opposed to the light strand (C-rich) for the other cell types (Figure 1A). One possible reason for different rNMP-embedment patterns in the liver tissue samples is that the liver tissue cells may have different abundances for each type of rNMP in their nucleotide pool compared to other cell types. Among the strongly conserved CrA, ArC, ArG and CrU patterns of yeast mtDNA, only CrU and CrA are found in hmtDNA and not in all cell types. Interestingly, the rNMP patterns found in hmtDNA are more similar to those found in mtDNA of fission yeast than to those of budding yeast, with a strong CrC pattern (21). Since RER with RNase H2 does not work to remove rNMPs in hmtDNA, the rNMP-embedment pattern in hmtDNA may mainly reflect the capacity of human Pol γ to accommodate rNMPs in its active site, which is considered the main source of rNMPs in mtDNA (12). Potentially, other factors, like transcription and/or activity of specific enzymes like topoisomerases may also influence the patterns of rNMPs observed in hmtDNA. Besides, the embedded rNMPs in hmtDNA, if present in stretches, may be removed by RNase H1 (15), mutations of which have been shown to impair hmtDNA replication (67). Thus, it would be valuable to also compare the rNMP-embedment patterns and features in wild-type with those obtained in mutant RNase H1 cells.

In conclusion, our study has revealed numerous features of rNMPs embedded in hmtDNA that are mainly distinct from those found in yeast mtDNA. The results also link features of the hmtDNA rNMPs to specific characteristics of hmtDNA, Pol γ rNMP-incorporation preference, hmtDNA replication, and transcription. The findings are opening new directions of investigation to better understand the biology of hmtDNA, as well as the cryptic roles of rNMPs embedded in genomic DNA.

## Supplementary Material

gkad1204_Supplemental_Files

## Data Availability

The DNA-seq and ribose-seq datasets for all hmtDNA libraries are available in NCBI’s BioProject via accession number ‘PRJNA941970’. The ribose-seq dataset for budding yeast *S. cerevisiae* is available in NCBI’s BioProject via accession number ‘PRJNA613920’. The emRiboSeq datasets analyzed are available in NCBI’s Gene Expression Omnibus via accession number ‘GSE64521’. Codes generated and used for analysis are available under the MIT License on Zenodo (https://doi.org/10.5281/zenodo.10211459). The scripts used to remove rNMPs at restriction enzyme locations are available under the GNU GPL V3.0 license on Zenodo (https://doi.org/10.5281/zenodo.8121711). The scripts used for rNMP mismatch removal are available under the GNU GPL V3.0 License on Zenodo (https://doi.org/10.5281/zenodo.10211459). The scripts used for locating rNMP hotspots and high-frequency rNMP embedment positions are available under the MIT License on Zenodo (https://doi.org/10.5281/zenodo.8152071).
