# Supplementary material for: Light-strand bias and enriched zones of embedded ribonucleotides are associated with DNA replication and transcription in the human-mitochondrial genome
Source: Nucleic Acids Res. 2023 Dec 20;52(3):1207–25. doi: 10.1093/nar/gkad1204 (PMC10853789; doi:10.1093/nar/gkad1204)

Supplementary Table S1 rNMPs found in ribose-seq libraries of hmtDNA.
List of mitochondrial ribose-seq libraries constructed in this study with corresponding data information and an indication of cell type, genotype, library name, fragmentation method, number of rNMPs, % rA, rC, rG, and rU with mean and standard deviation for each cell type, barcode, and number of cycles in PCR 1 and PCR 2 of the ribose-seq protocol.

Supplementary Table S2 Oligonucleotides used in this study.
The name, length, and sequence of oligonucleotides used in this study are presented. All bold letters in the PCR primers indicate the specific sequence of the index used in sequencing. P and Am indicate end modifications of phosphate and amino modifier, respectively. All oligonucleotides were desalted, except those marked with an asterisk (*), which were HPLC purified. All oligonucleotides were synthesized by Integrated DNA Technologies.

Supplementary Table S3 Common rNMP-enriched zones in hmtDNA.
Table lists the name, strand, start, end, mean, and median enrichment factor of all common REZs in hmtDNA (See Material and Methods), and all libraries containing these common REZs.

Supplementary Table S4 rNMP hotspots in all libraries and each subset in hmtDNA.
Table represents rNMP hotspots in all libraries and each subset $CD4^{+}T$, DLTB, hESC-H9, HEK293T, and HEK293T RNH2A KO. The location, strand, and rNMP with dNMPs in +/- 3 nt locations of each hotspot are indicated in the table. Median EF represents the median value of the enrichment factor found in each library.

Supplementary Table S5 Statistical test for preferred rNMP composition and pattern.
Mann-Whitney *U* test for rNMP composition and preferred dinucleotide and trinucleotide patterns in hmtDNA and *S. cerevisiae* mtDNA. Right-sided Mann-Whitney *U* tests are performed on the normalized frequencies versus the expectation value (0.25 for composition and dinucleotide pattern, 0.125 for trinucleotide pattern) for each cell type and all cell types combined. Cells with bold font show statistically significant results with a P-value < 0.05, which means the pattern is preferred.

Supplementary Table S6 Statistical test for rNMP-embedment composition and pattern bias between the two strands.
Mann-Whitney *U* test for rNMP composition and preferred dinucleotide and trinucleotide patterns between light and heavy strands in hmtDNA and between forward and reverse strands in *S. cerevisiae*. Two-tailed paired Mann-Whitney *U* tests are performed on the normalized frequencies between both strands. Cells with bold font show statistically significant results with a P-value < 0.05, which means the pattern is biased between the two strands.

Supplementary Table S7 Maximum rNMP count in each library.
The maximum rNMP counts at the single-nucleotide position on each strand are calculated. “Positive Maxcount” and “Negative Maxcount" in the table are the maximum rNMPs count in the light strand and heavy strand.

Supplementary Figure S1 Embedded rNMPs are prevalent on the reverse strand of *S. cerevisiae* mtDNA.

(**A**) Bar graph showing the percentage of embedded rNMPs on the light (red bars) and heavy (blue bars) strands in yeast mtDNA. The green vertical line separates RNH201 WT and KO (*rnh201*) libraries. Two-tailed Mann-Whitney *U* test is performed to check the significance of the light/heavy strand bias. *: $0.01 < P < 0.05$; **: $0.001 < P < 0.01$; ***: $0.0001 < P < 0.001$; ****: $0.00001 < P < 0.0001$. (**B**) rNMP frequency at each nucleotide on the forward (top red spikes) and reverse (bottom blue spikes) strands of yeast mtDNA of ribose-seq (FS) and emRiboSeq (EM) libraries. Examples of consistent enrichment of rNMP on the light and heavy strands are indicated by red and blue arrows.

Supplementary Figure S2 rNMP distribution on both strands of hmtDNA.

The rNMP embedment at each nucleotide on the light (outer-circle red spikes) and heavy (inner-circle blue spikes) strands of hmtDNA in each library. The maximum spike heights represent the half of maximum counts of rNMPs of both light and heavy strands indicated in **Supplementary Table S7**.

Supplementary Figure S3 rNMP-enriched zones in the hmtDNA control region.

rNMP embedment probability per base (PPB) in the hmtDNA control region for each cell category. The upper part of the figure is the rNMP PPB on the light strand and the lower one is on the heavy strand. The OriH region is shown in green color and the rest of the control region is shown in orange color. The standard deviation is indicated by the shadow.

Supplementary Figure S4 Embedded rNMPs inhibit the binding activity of Pol γ and TWINKLE.

The rNMP PPB on the light and heavy strand of the control region in all RNH2A WT libraries with chromatin immunoprecipitation-sequencing (ChIP-seq) data of Pol γ and TWINKLE (47). The blue and red bent arrows indicate the heavy-strand promoter (HSP) and light-strand promoter (LSP), respectively. Around the high PPB region, there is a lower ChIP-seq signal compared to neighborhood regions.

Supplementary Figure S5 The rNMP-embedment frequency does not correlate with CDS length on the template strand and non-template strand of non-coding genes.

(**A**) rNMP-embedment probability per base (PPB) on the template strand of each coding sequence in each library. The PPB measures the normalized rNMP-embedment frequency at each base (See Material and Methods). Each row represents a CDS, which is sorted by descending gene size. Each column represents an rNMP library. There are three control libraries with artificial rNMP embedment generated *in silico* on the right (See Material and Methods). (**B**) Linear regression plot showing no correlation between the rNMP enrichment factor and gene size on the template strand. Each data point represents the rNMP-enrichment factor on the template strand of a particular gene in an rNMP library of a given cell type. The rNMP-enrichment factors are calculated as described in Methods. Spearman's r and p-value are marked in the plot. Cell types with $r > 0.5$ have a strong positive correlation between the rNMP-enrichment factor and gene size. The 95% confidential interval is marked as the blue shadow region around the line. (**C**) Linear regression plot on both non-template and template strands for other cell categories. (**D**) rNTP-incorporation probability per base (PPB) on the non-template strand of each non-coding gene in each library.

Supplementary Figure S6 The rNMP-embedment frequency does not correlate with CDS length in *S. cerevisiae* mtDNA.
rNMP-embedment probability per base (PPB) on the (**A**) non-template strand and (**B**) template strand of each coding sequence in each library of *S. cerevisiae* mtDNA. The PPB measures the normalized rNTP-incorporation frequency at each nucleotide (See Material and Methods). Each row represents a gene, which is sorted by descending gene size. Each column represents an rNMP library.

Supplementary Figure S7 rNMP composition in mtDNA of each library.
Heatmap analyses with the normalized frequency of incorporated rNMP (R: rA, rC, rG, or rU) on (**A**) both strands, (**B**) light and heavy strand of hmtDNA in all libraries.

Supplementary Figure S8 Consensus analysis for high-frequency rNMP location motifs in hmtDNA of the different cell types.
Sequence-motif plots for high-frequency rNMP locations (Top 1% sites of most abundant embedded rNMPs, see Material and Methods). Position 0 on the x-axis represents the rNMP site, - and + positions represent upstream and downstream dNMPs, respectively. The y-axis shows the level of sequence conservation, represented in bits. The cell type, genotype, library name, and the number of rNMP sites are included below each plot.

Supplementary Figure S9 rNMP composition in *S. cerevisiae*.
(**A, B**) Bar plot showing the normalized frequency of the four rNMP types on the (**A**) forward strand and the (**B**) reverse strand of yeast mtDNA. The normalized frequency is calculated as described in the Material and Methods section. Types of rNMPs and their corresponding colors are listed in the color legend on the right top. (**C, D**) Heatmap analyses with the normalized frequency of incorporated rNMP (R: rA, rC, rG, or rU) on the (**C**) forward strand and the (**D**) reverse strand of yeast mtDNA in all libraries. The vertical green line separates the cell types with RNH2A WT and with RNH2A KO genotype.

Supplementary Figure S10 Relative rNTP abundance in different human cell types.
rNTP pool to dNTP pool ratios in total cells of CD4^{+}T, hESC-H9, HEK293T WT RNH2A, and RNH2A-KO. dNTP and rNTP concentration (pmol/million cells) for activated CD4^{+}T cells were collected from a previous study having 4 replicates, and the precalculated average was used to represent dNTP/rNTP ratios in this figure (55). Data on the rest of the cell lines were collected for this study and nucleotide contents were quantified using Thermo TSQ Quantiva Lc-MS/MS with Kinetex EVO-C18 column. Pmol/million cells were calculated for rNTPs and dNTPs for all four replicates to calculate the ratios in this figure.

Supplementary Figure S11 Downstream dNMP is not associated with incorporated rNMP.
(**A**) Heatmap analyses with the normalized frequency of dinucleotides composed of the incorporated rNMP (R: rA, rC, rG, or rU) and its downstream dNMP neighbor (N: dA, dC, dG, or dT) (RN) in the hmtDNA. The rNMP position in the dinucleotide is shown in red at the top left of the heatmap. Each column of the heatmap shows the results of an rNMP library. The vertical, green lines separate different cell types. The color scale is shown in the top right corner of the figure.

Supplementary Figure S12 Biased rNTP-incorporation dinucleotide patterns between the light and heavy strands of hmtDNA.
Heatmap analyses with the normalized frequency of dinucleotides composed of the incorporated rNMP (R: rA, rC, rG, or rU) and its upstream dNMP neighbor (N: dA, dC, dG, or dT) (NR) in the (**A**) light and (**B**) heavy strand of hmtDNA. The rNMP position in the dinucleotide is shown in red at the top left of the heatmap. Each column of the heatmap shows the results of an rNMP library. The vertical, green lines separate different cell types. The color scale is shown in the top right corner of the figure.

Supplementary Figure S13 Similar rNTP-incorporation dinucleotide patterns between the forward and reverse strands in *S. cerevisiae* mtDNA.
Heatmap analyses with the normalized frequency of dinucleotides composed of the incorporated rNMP (R: rA, rC, rG, or rU) and its upstream dNMP neighbor (N: dA, dC, dG, or dT) (NR) in the (**A**) forward and (**B**) reverse strand of yeast mtDNA. The rNMP position in the dinucleotide is shown in red at the top left of the heatmap. Each column of the heatmap shows the results of an rNMP library. The vertical, green lines separate different RNase H2 genotypes. The color scale is shown in the top right corner of the figure.

Supplementary Figure S14 Preferred trinucleotide of embedded rNMPs in hmtDNA.
Heatmap analyses with the normalized frequency of trinucleotides composed of the incorporated rNMP (R: rA, rC, rG, or rU) and its two upstream dNMP neighbors (N: dA, dC, dG, or dT) (NNR) in the (**A**) both strands, (**B**) light strand, and (**C**) heavy strand of hmtDNA. The vertical, green lines separate different cell types. The color scale is shown in the bottom right corner of the figure.

Supplementary Figure S15 Preferred trinucleotide of embedded rNMPs in budding yeast *S. cerevisiae* mtDNA.
Heatmap analyses with the normalized frequency of trinucleotides composed of the incorporated rNMP (R: rA, rC, rG, or rU) and its two upstream dNMP neighbors (N: dA, dC, dG, or dT) (NNR) in the (**A**) forward strand and (**B**) reverse strand of budding yeast *S. cerevisiae* mtDNA. The vertical, green lines separate different cell types. The color scale is shown in the bottom right corner of the figure.

Supplementary Figure S1

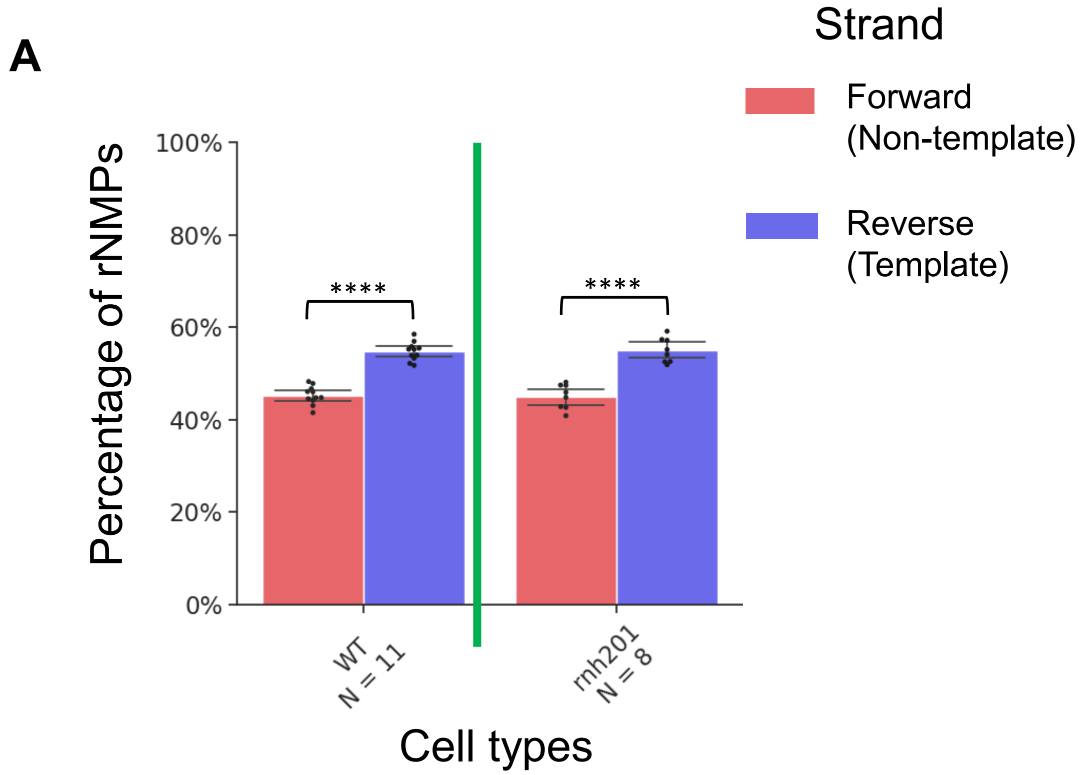

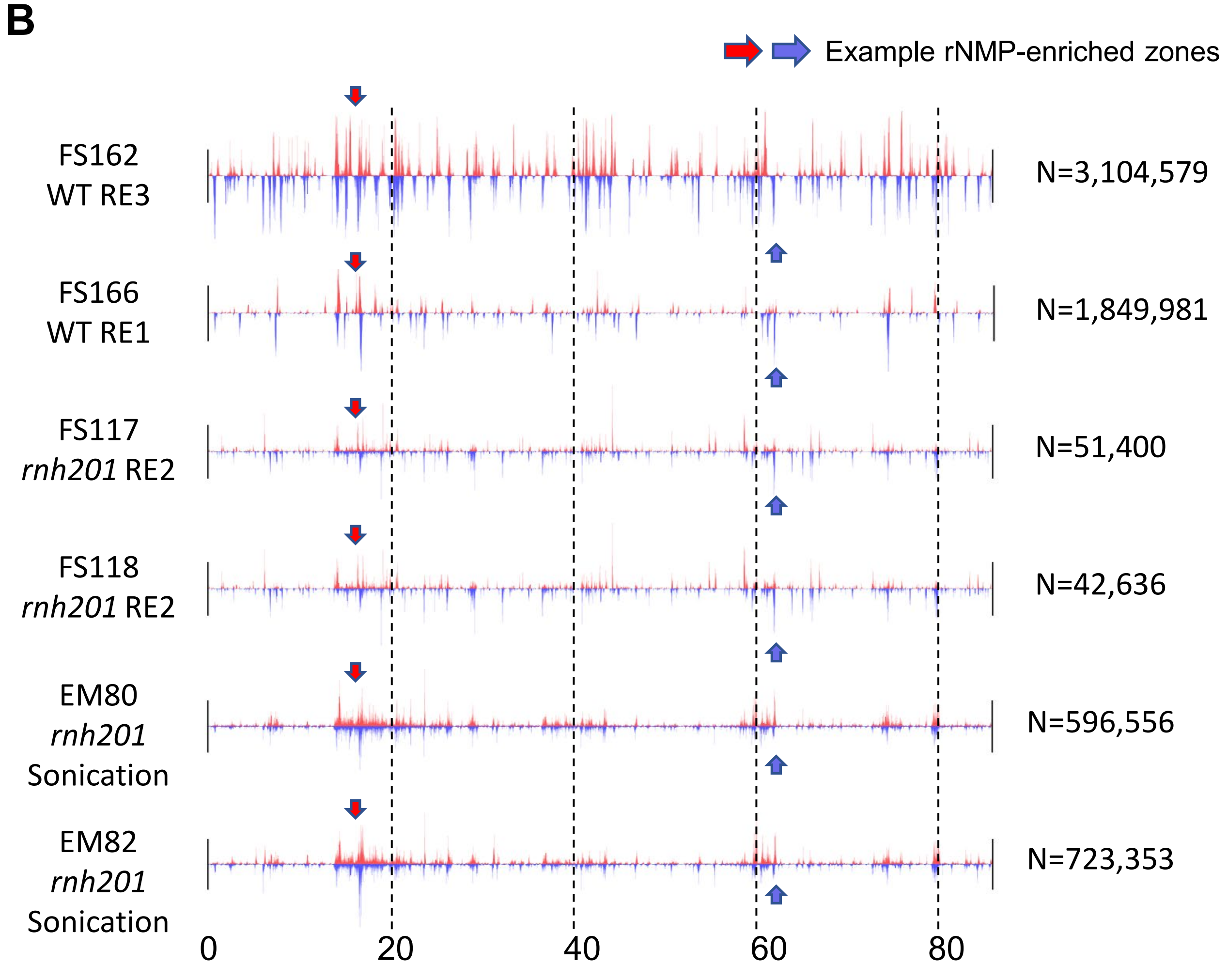

Supplementary Figure S2

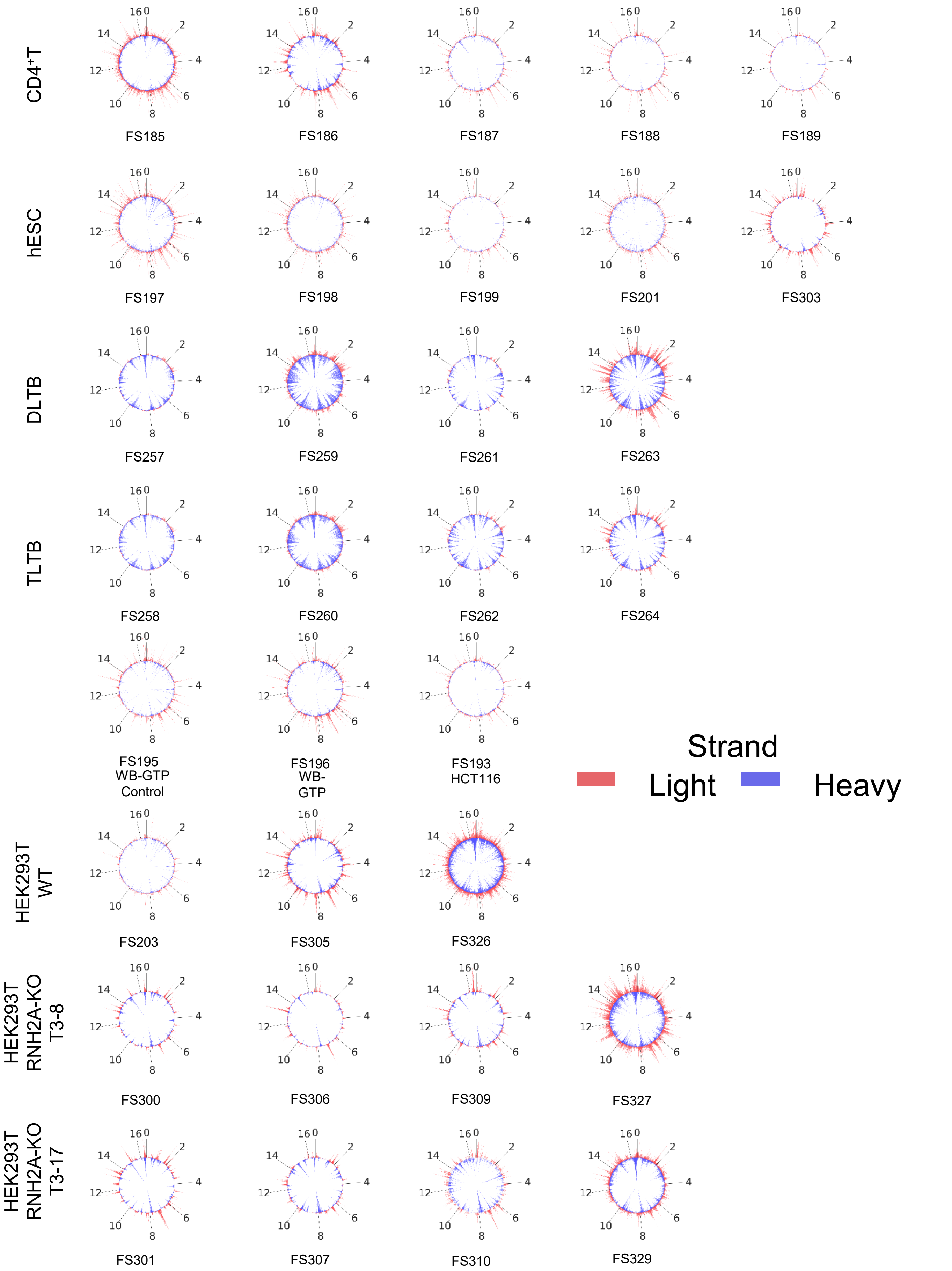

Supplementary Figure S3

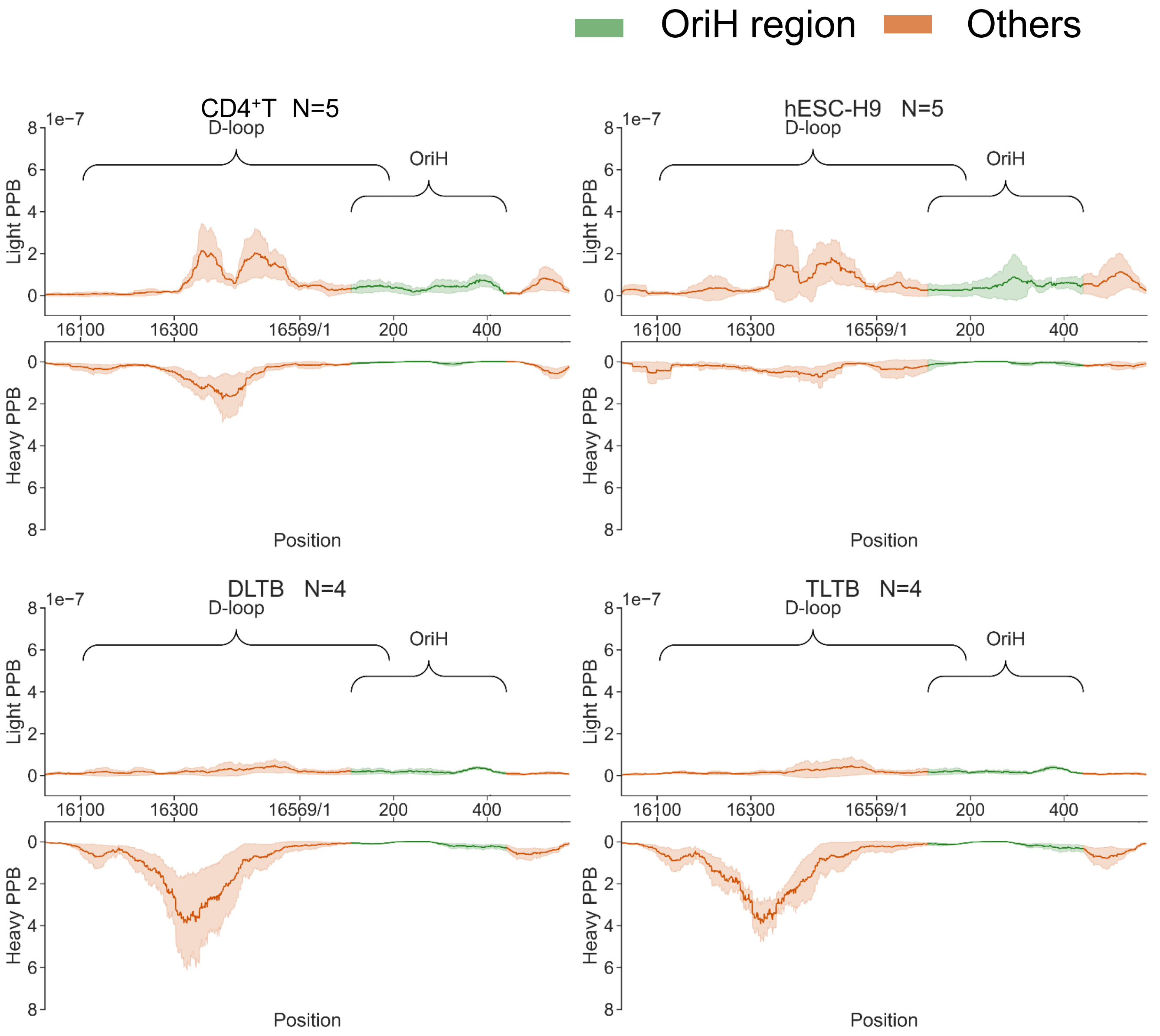

Supplementary Figure S3 continued

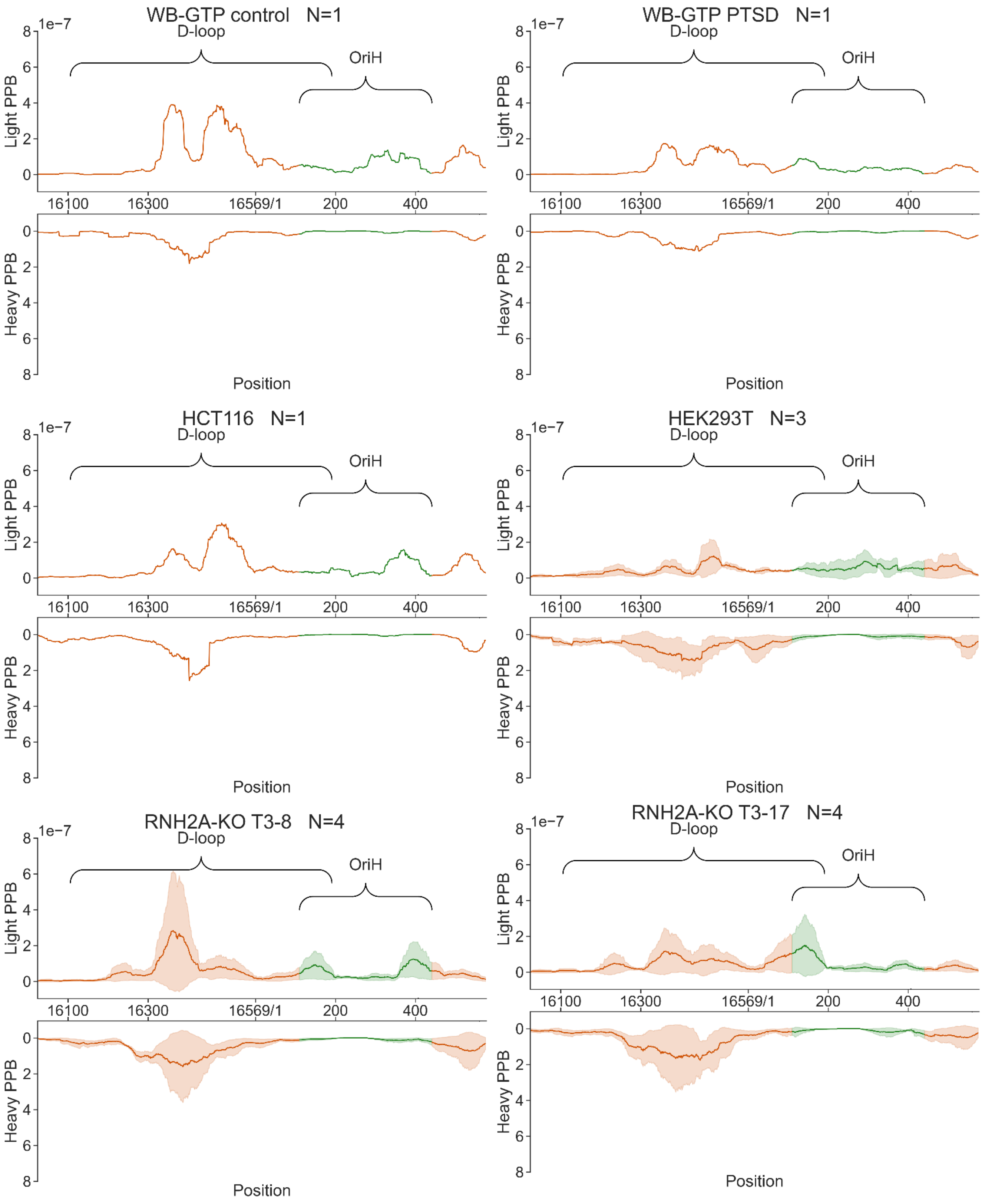

Supplementary Figure S4

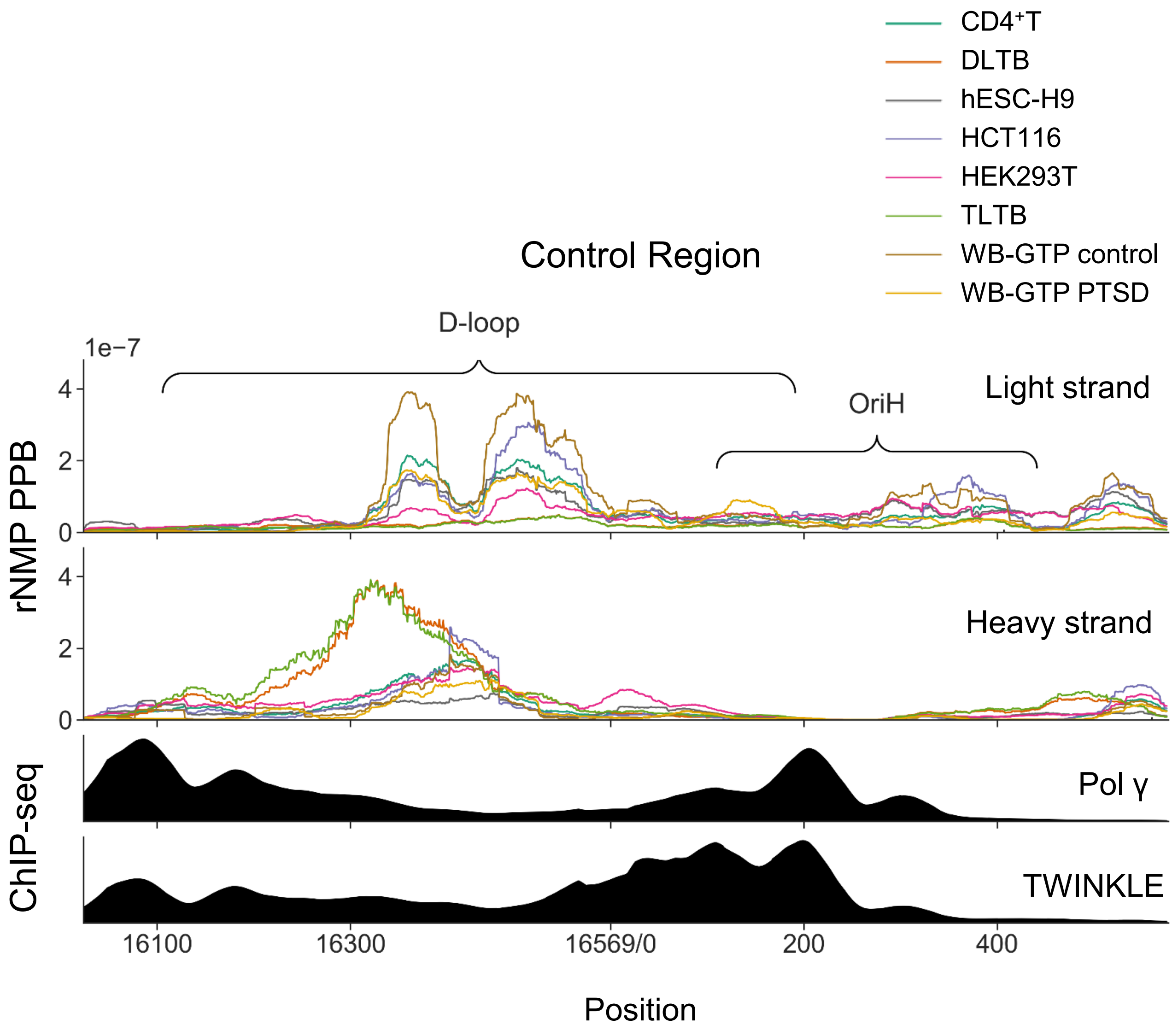

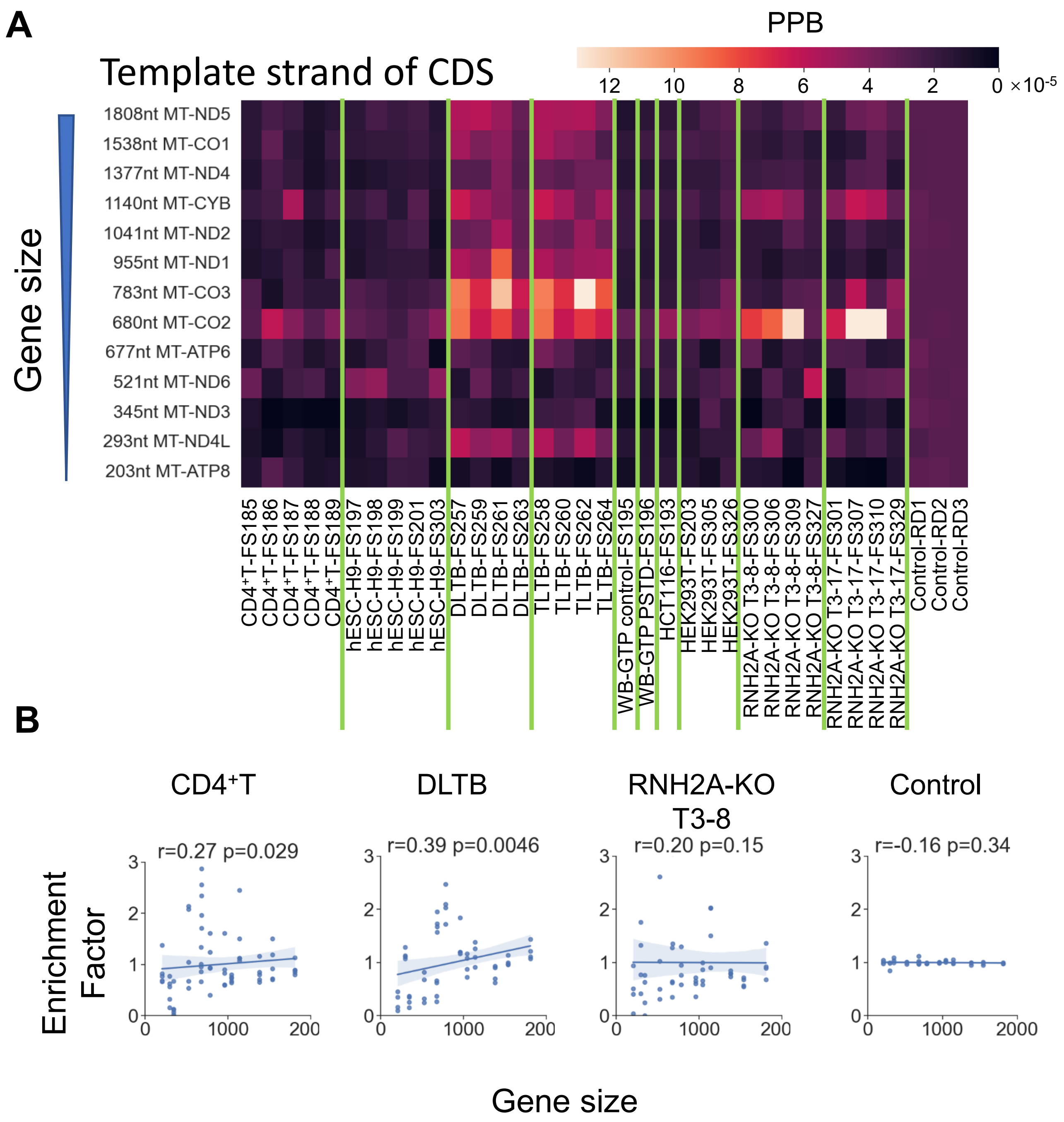
A
Template strand of CDS
PPB
12 10 8 6 4 2 0 ×10-5
Gene size
1808nt MT-ND5
1538nt MT-CO1
1377nt MT-ND4
1140nt MT-CYB
1041nt MT-ND2
955nt MT-ND1
783nt MT-CO3
680nt MT-CO2
677nt MT-ATP6
521nt MT-ND6
345nt MT-ND3
293nt MT-ND4L
203nt MT-ATP8
CD4+T-FS185
CD4+T-FS186
CD4+T-FS187
CD4+T-FS188
CD4+T-FS189
hESC-H9-FS197
hESC-H9-FS198
hESC-H9-FS199
hESC-H9-FS201
hESC-H9-FS303
DLTB-FS257
DLTB-FS259
DLTB-FS261
DLTB-FS263
TLTB-FS258
TLTB-FS260
TLTB-FS262
TLTB-FS264
WB-GTP control-FS195
WB-GTP PSTD-FS196
HCT116-FS193
HEK293T-FS203
HEK293T-FS305
HEK293T-FS326
RNH2A-KO T3-8-FS300
RNH2A-KO T3-8-FS306
RNH2A-KO T3-8-FS309
RNH2A-KO T3-8-FS327
RNH2A-KO T3-17-FS301
RNH2A-KO T3-17-FS307
RNH2A-KO T3-17-FS310
RNH2A-KO T3-17-FS329
Control-RD1
Control-RD2
Control-RD3
B
CD4+T
r=0.27 p=0.029
DLTB
r=0.39 p=0.0046
RNH2A-KO T3-8
r=0.20 p=0.15
Control
r=-0.16 p=0.34
Enrichment Factor
Gene size
0 1000 200
0 1000 2000

Supplementary Figure S5 continued

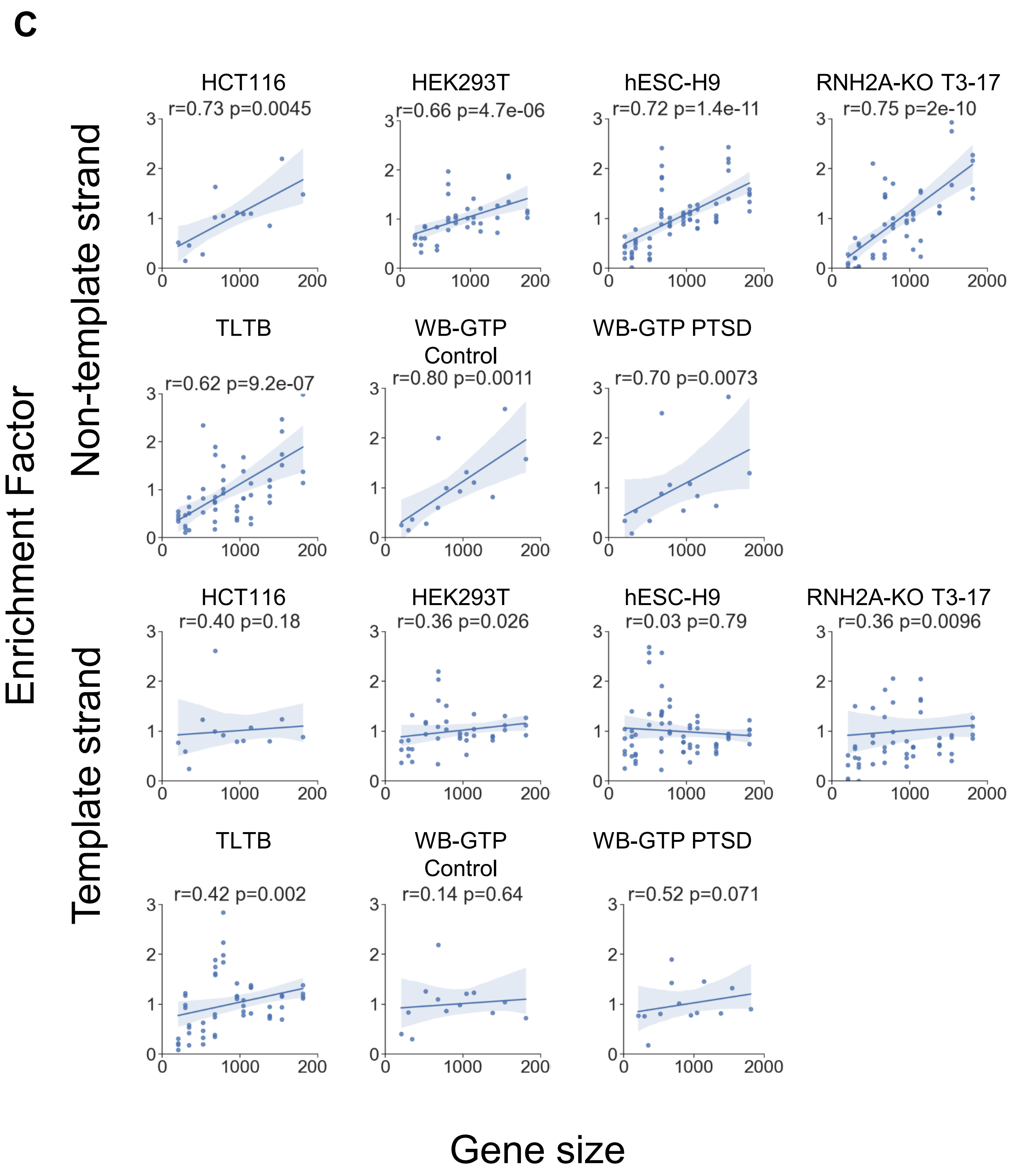

Supplementary Figure S5 continued

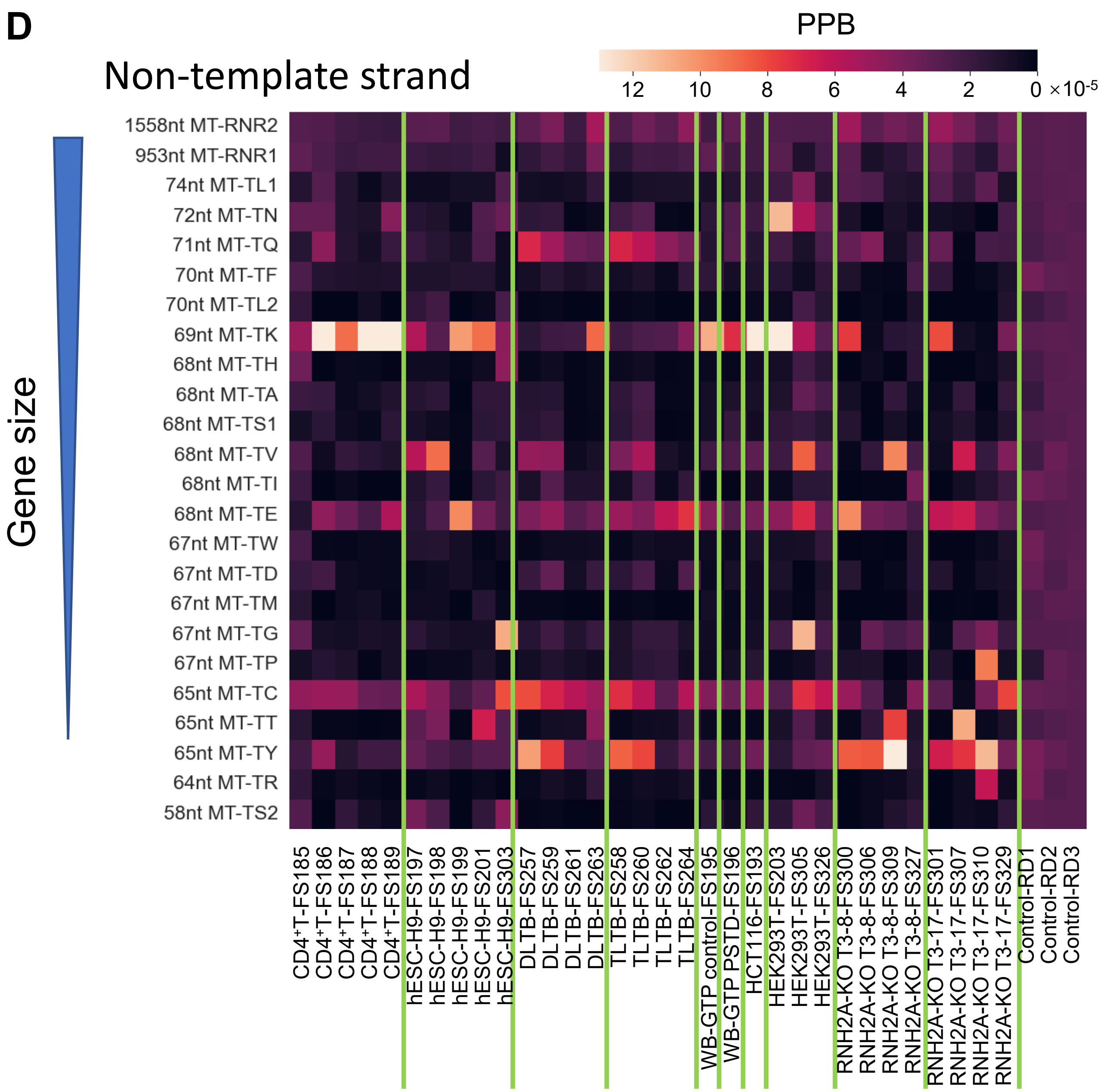

Supplementary Figure S6

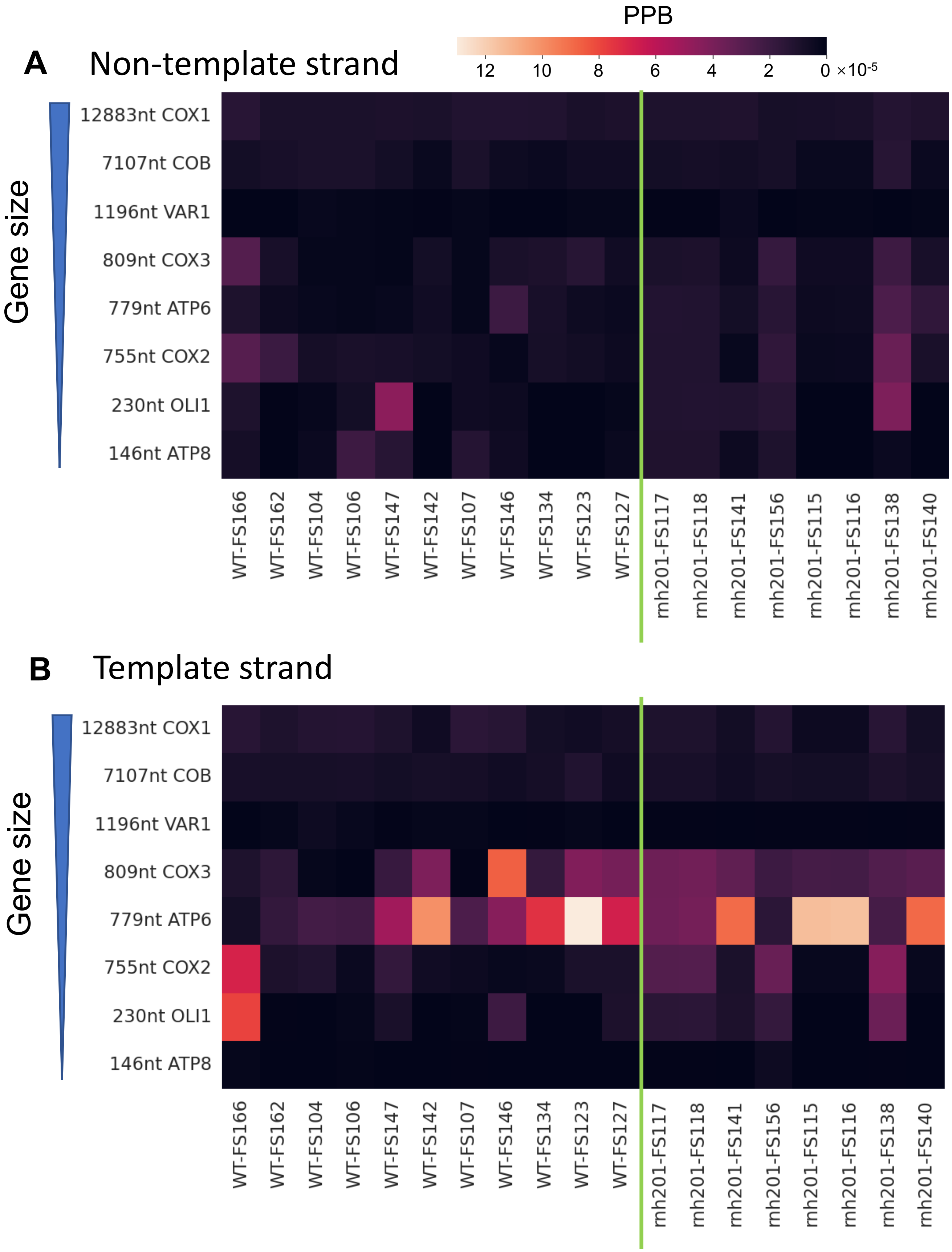

Supplementary Figure S7

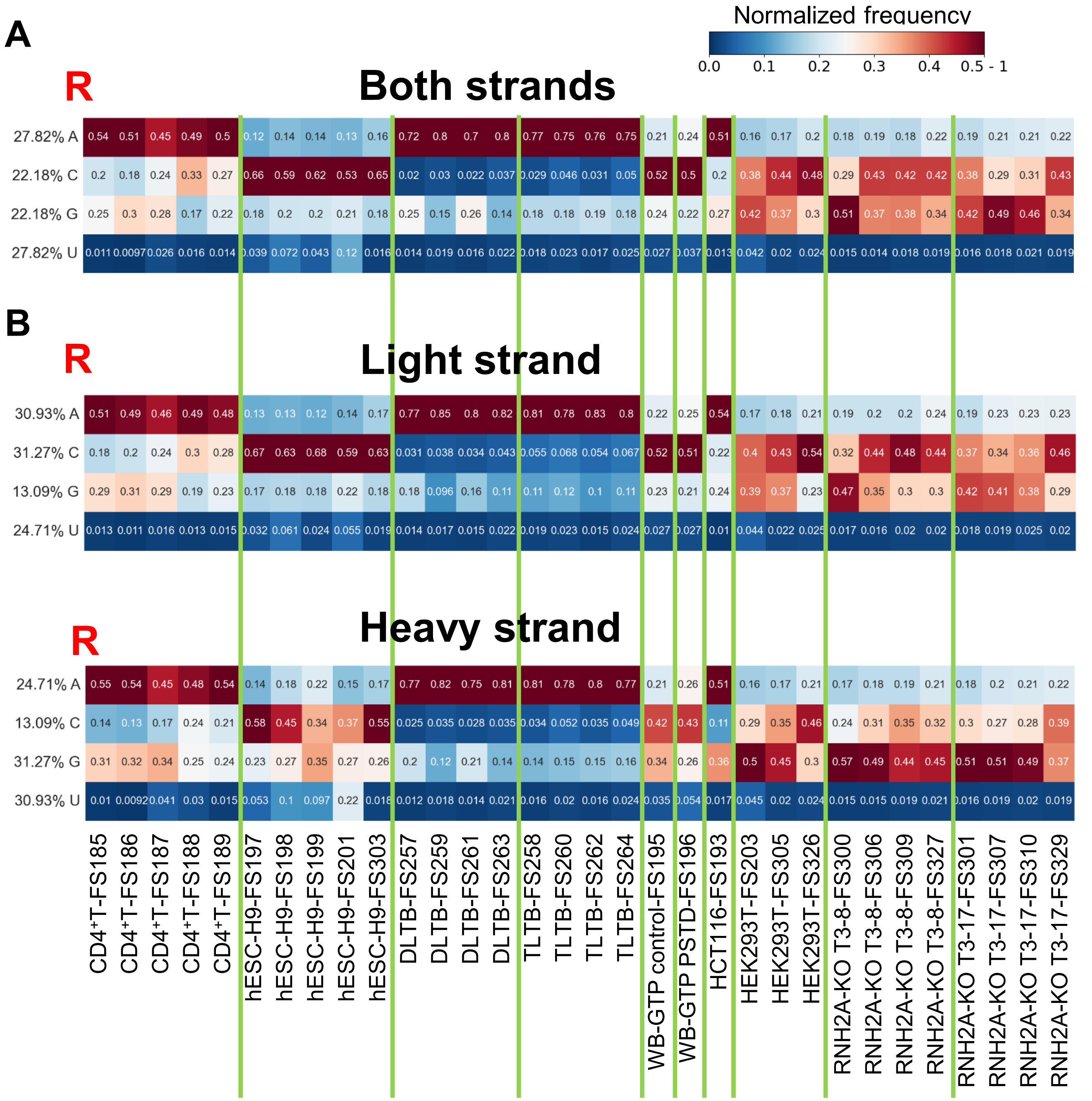

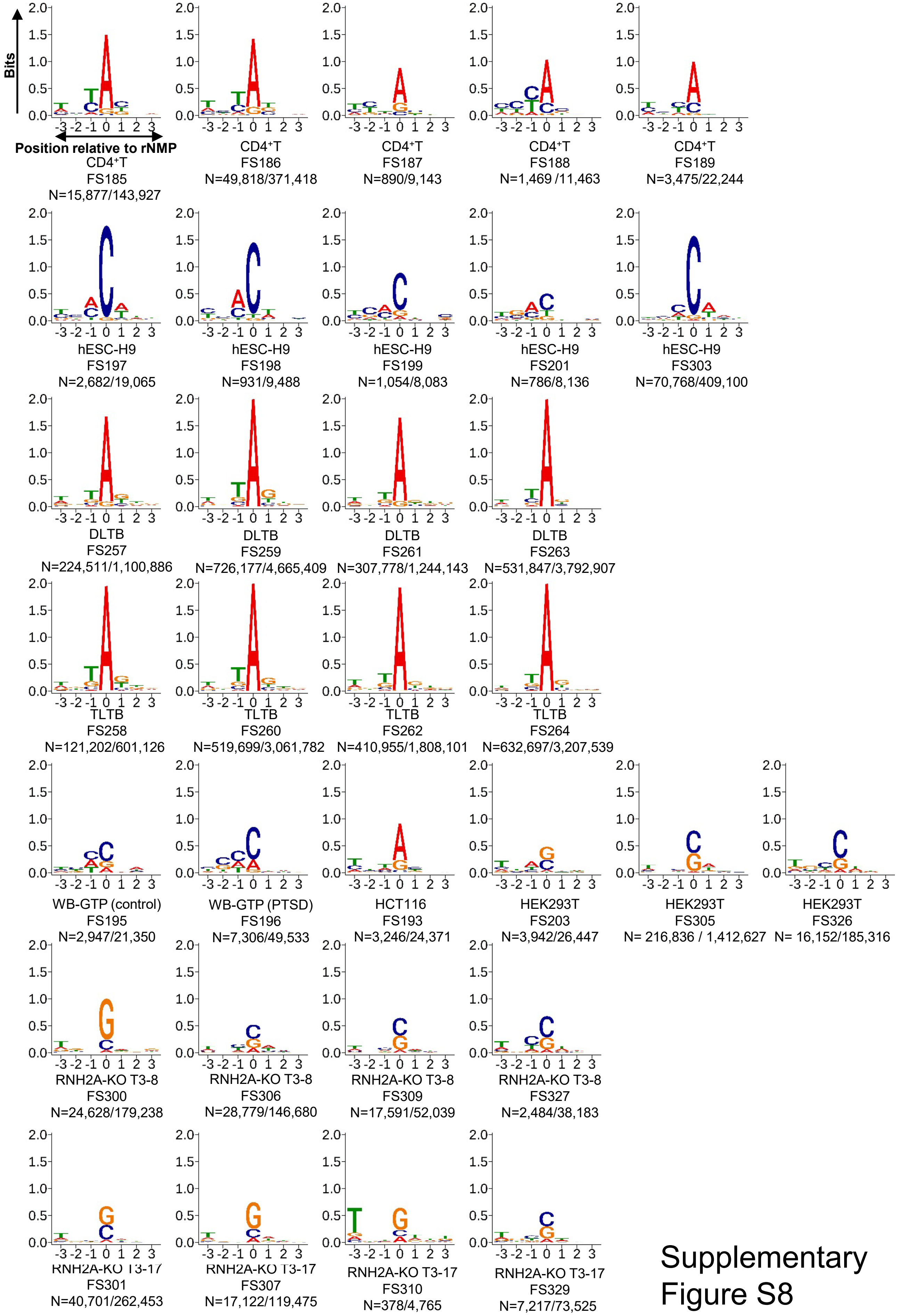

Supplementary Figure S8

Supplementary Figure S9

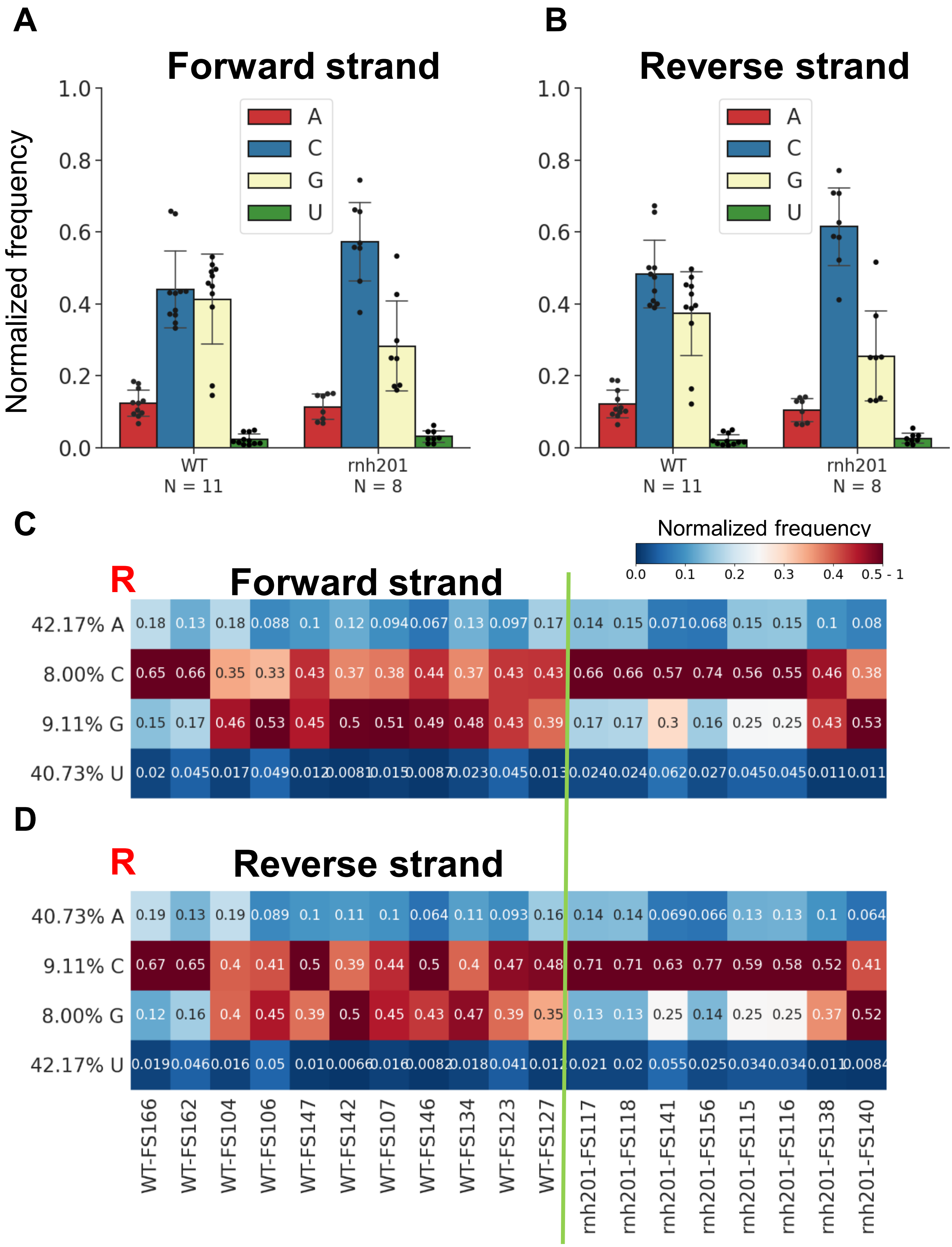

Supplementary Figure S10

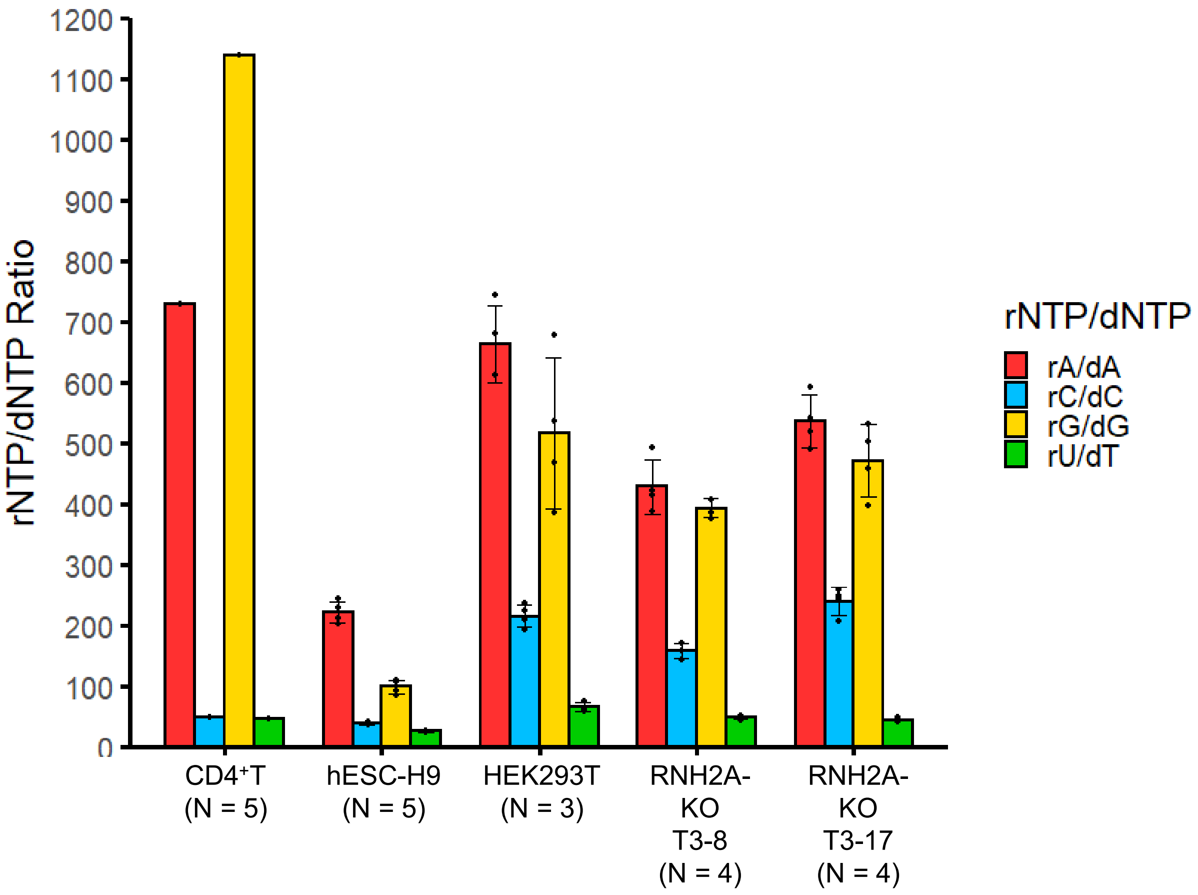

Supplementary Figure S11

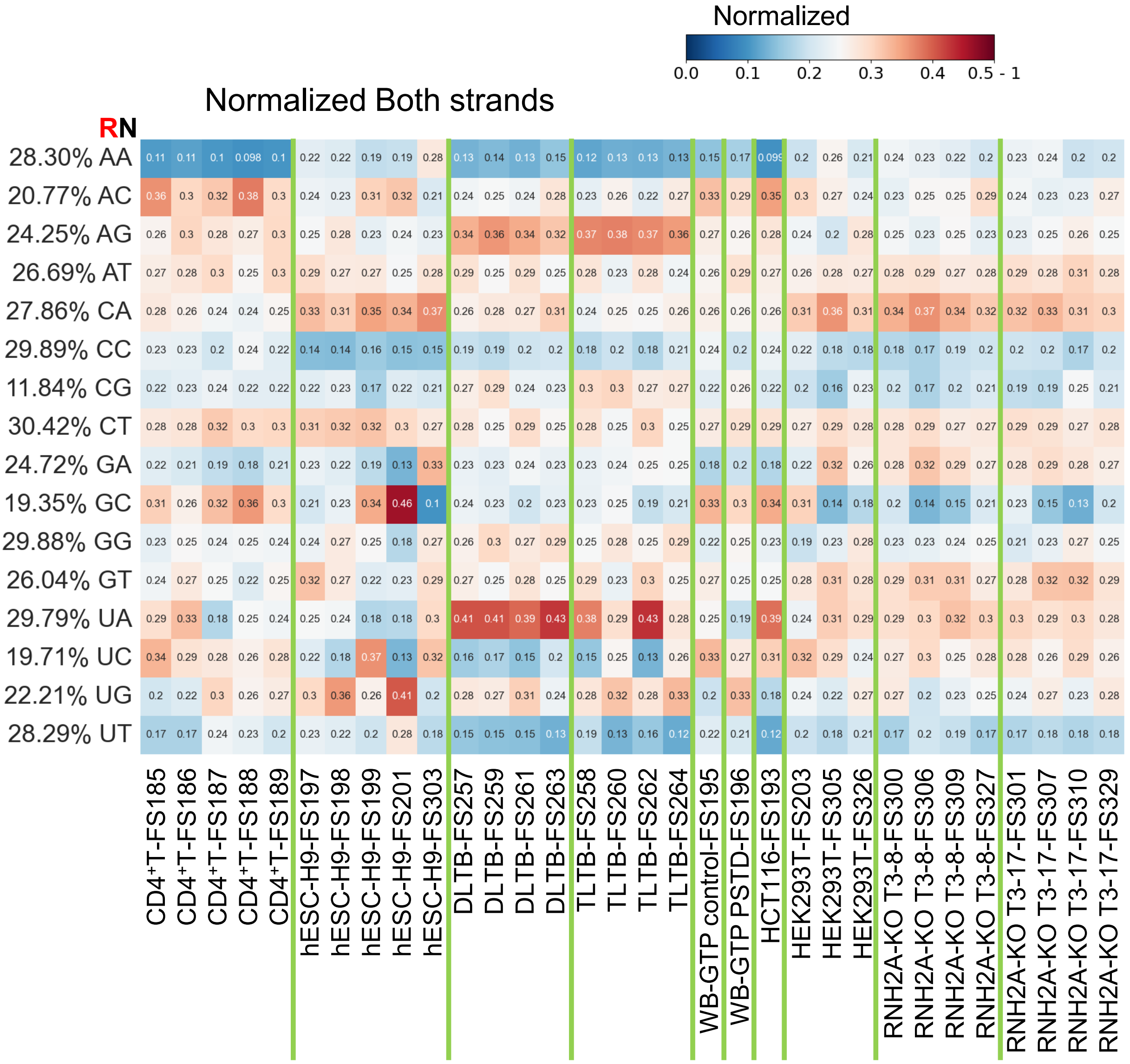

Supplementary Figure S12

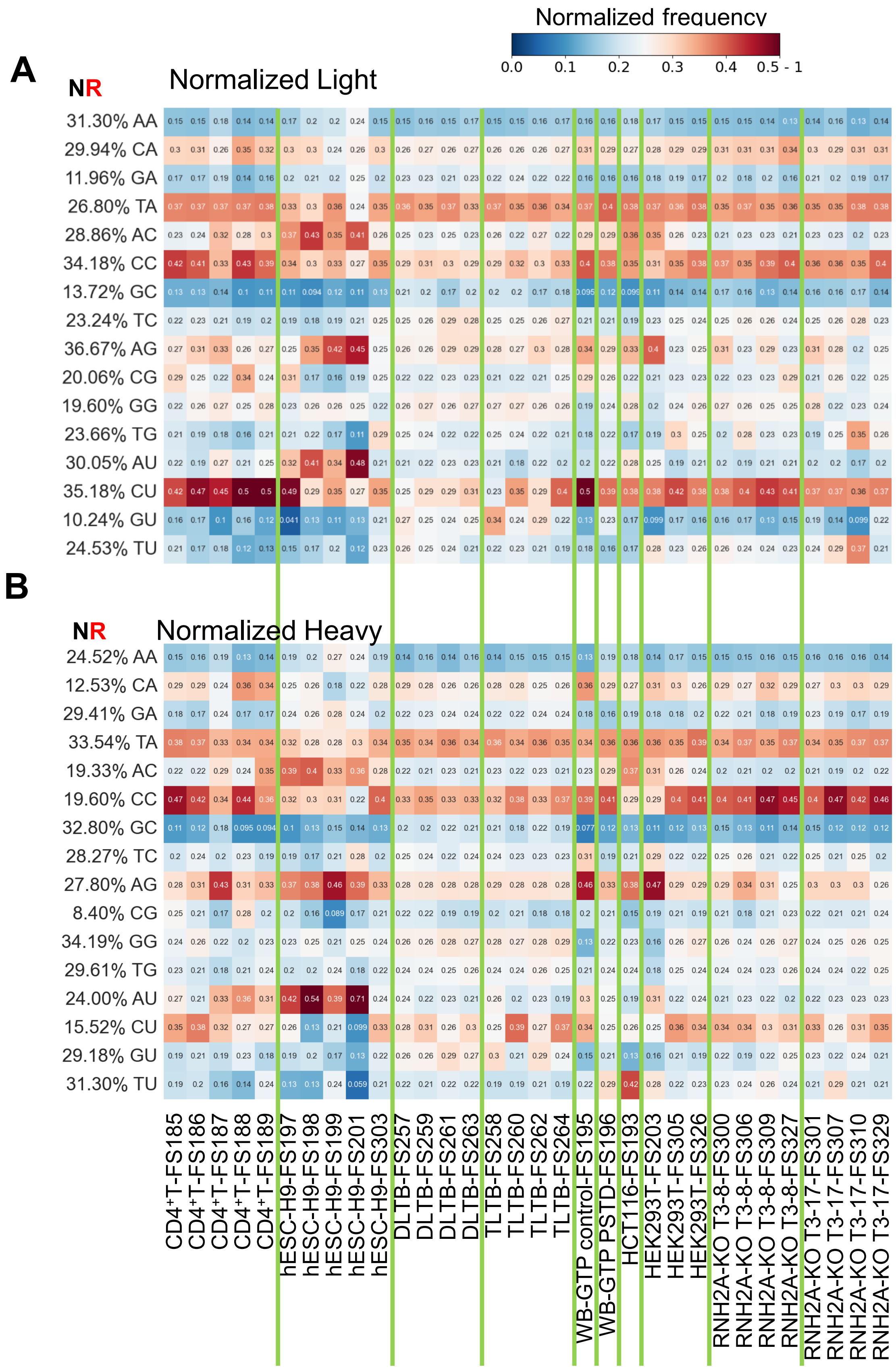

Supplementary Figure S13

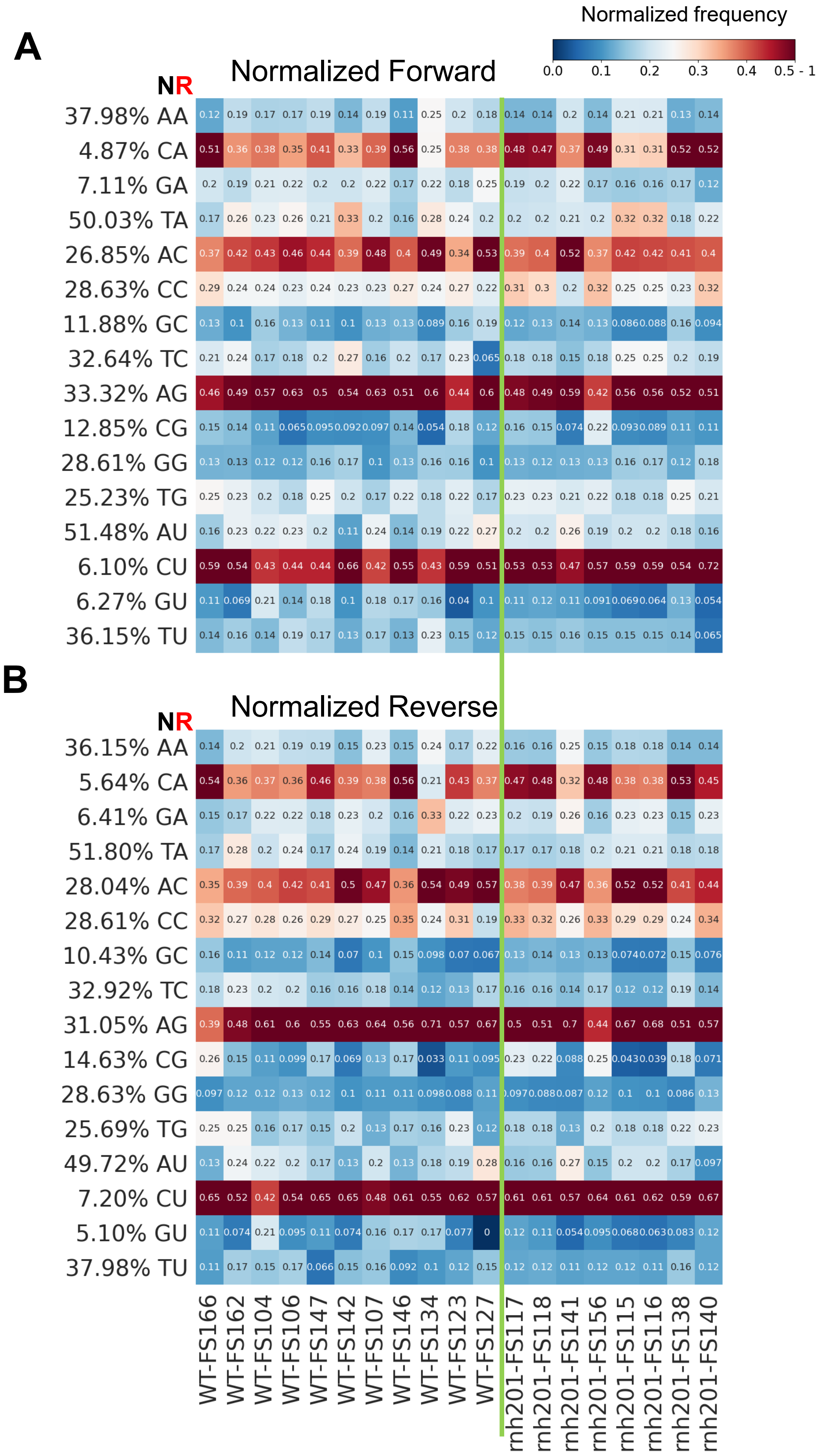

Supplementary Figure S14

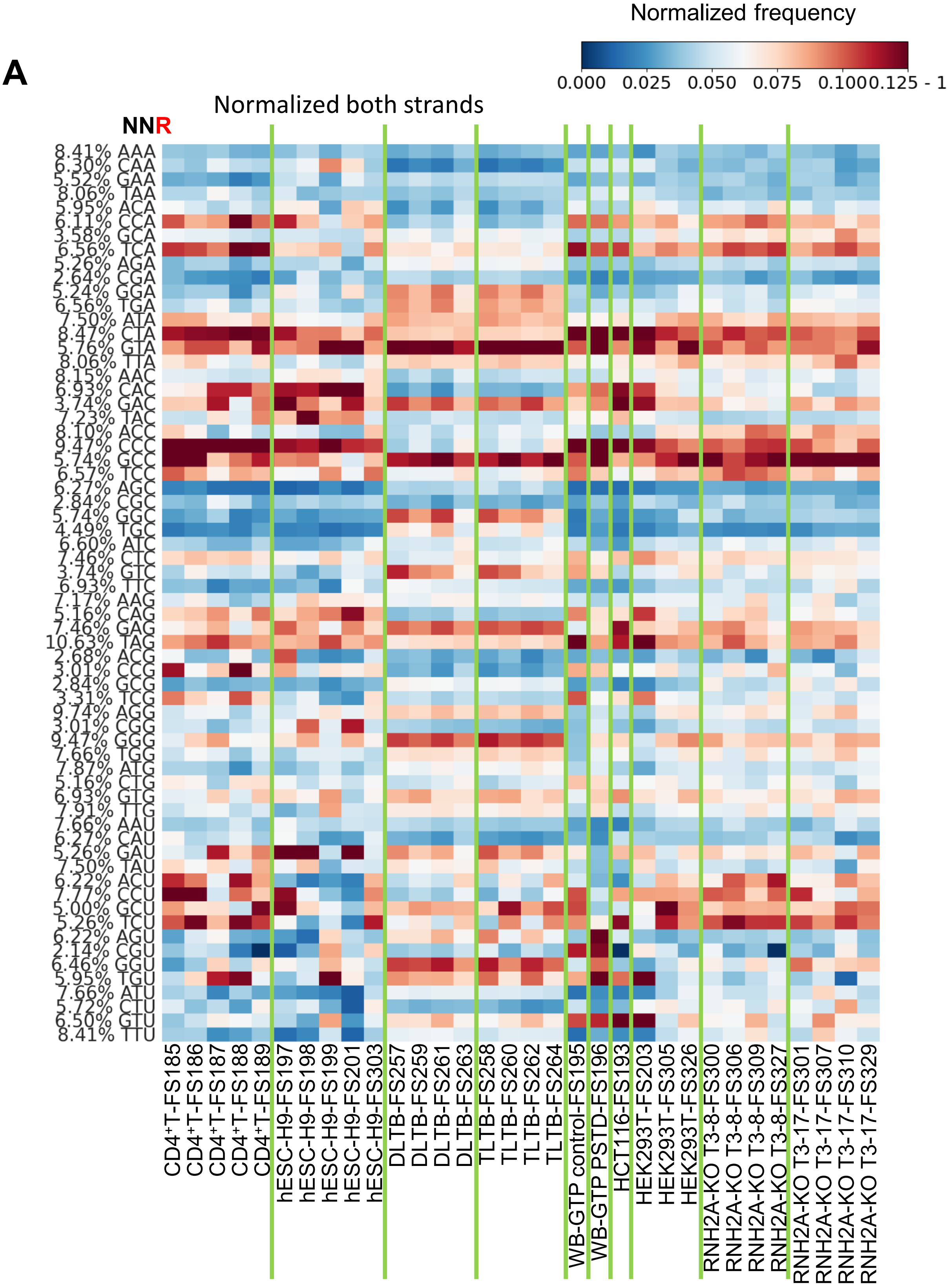

Supplementary Figure S14 continued

B

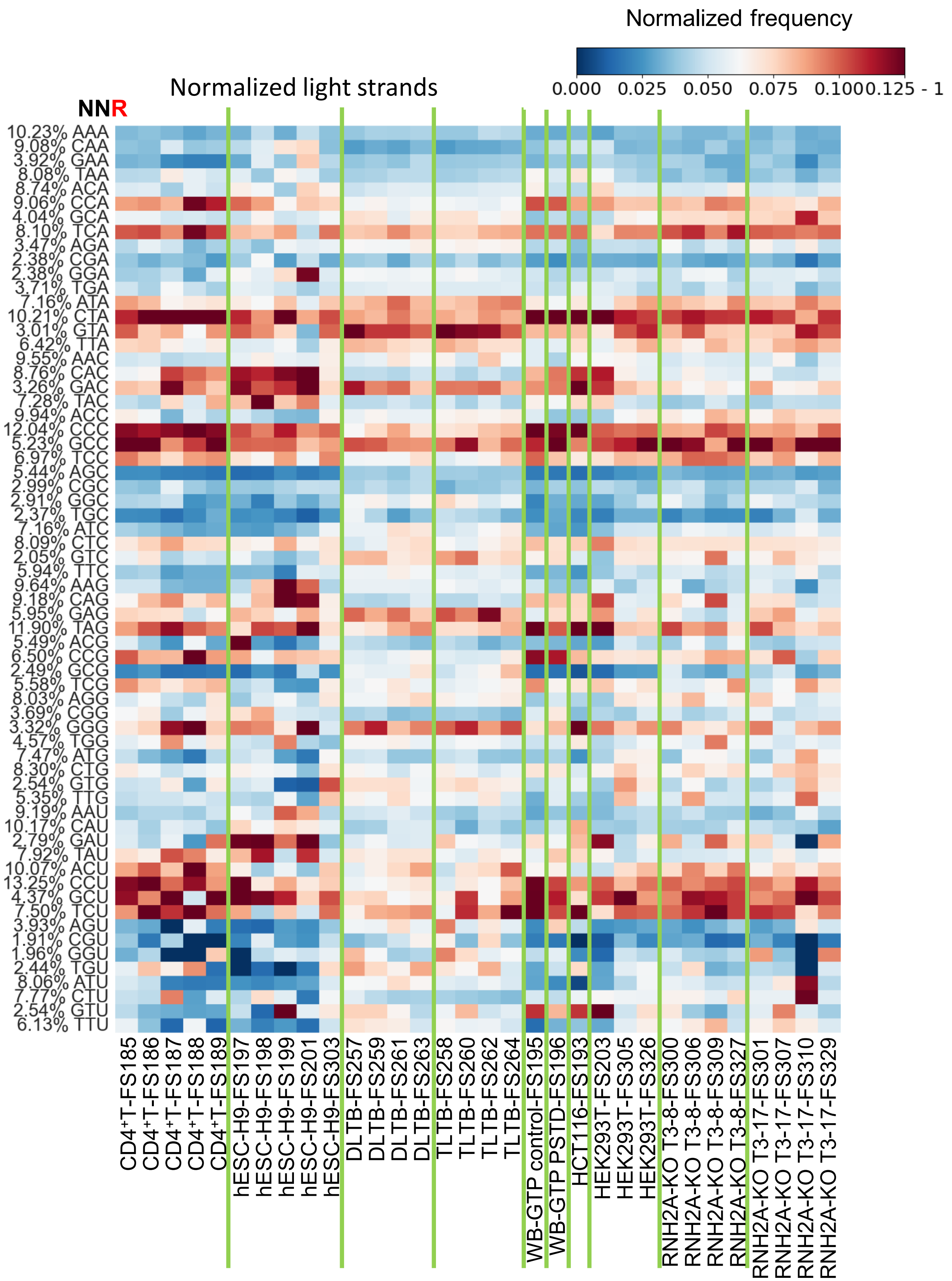

Supplementary Figure S14
continued

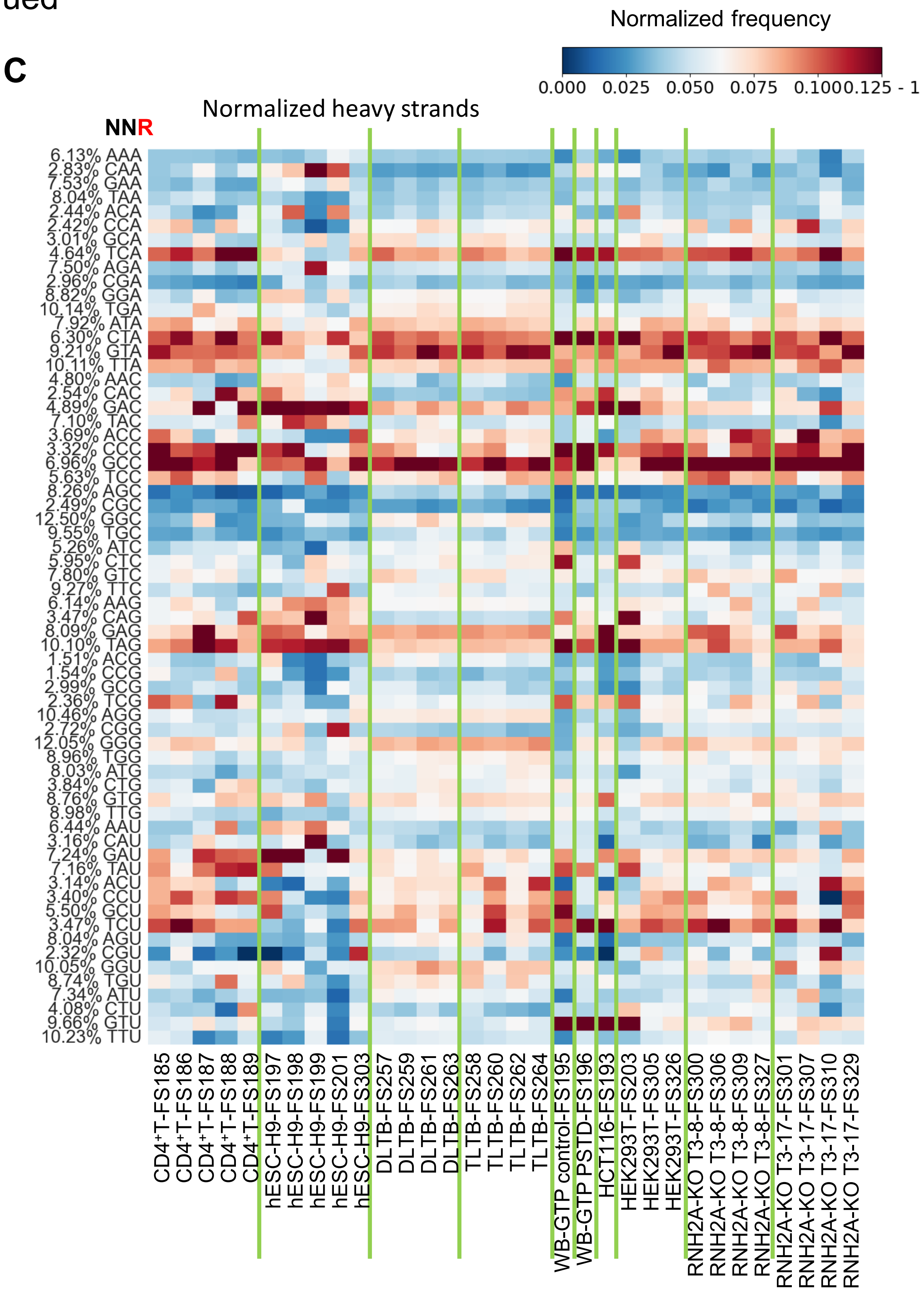

Supplementary Figure S15

A

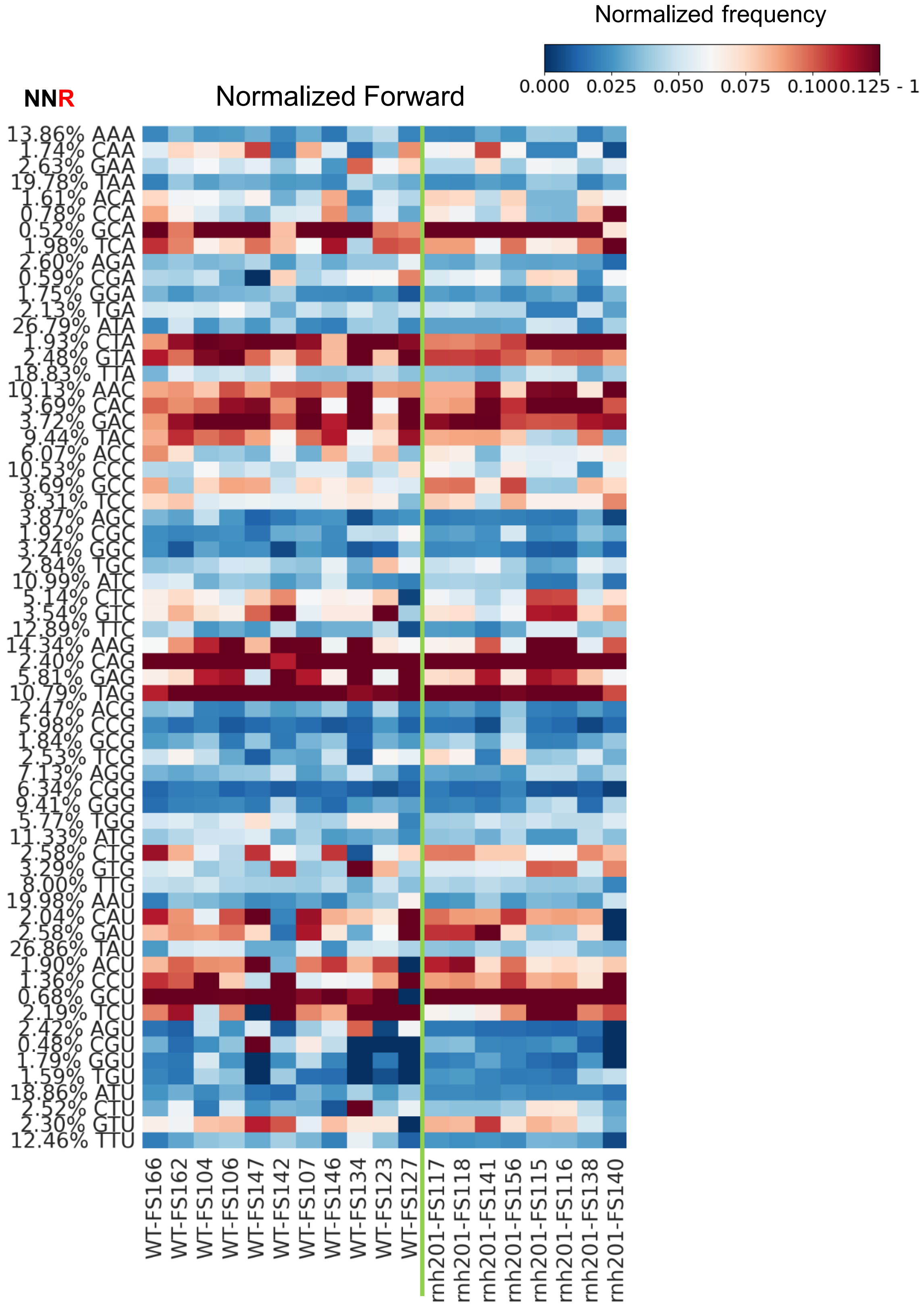

Supplementary Figure S15 continued

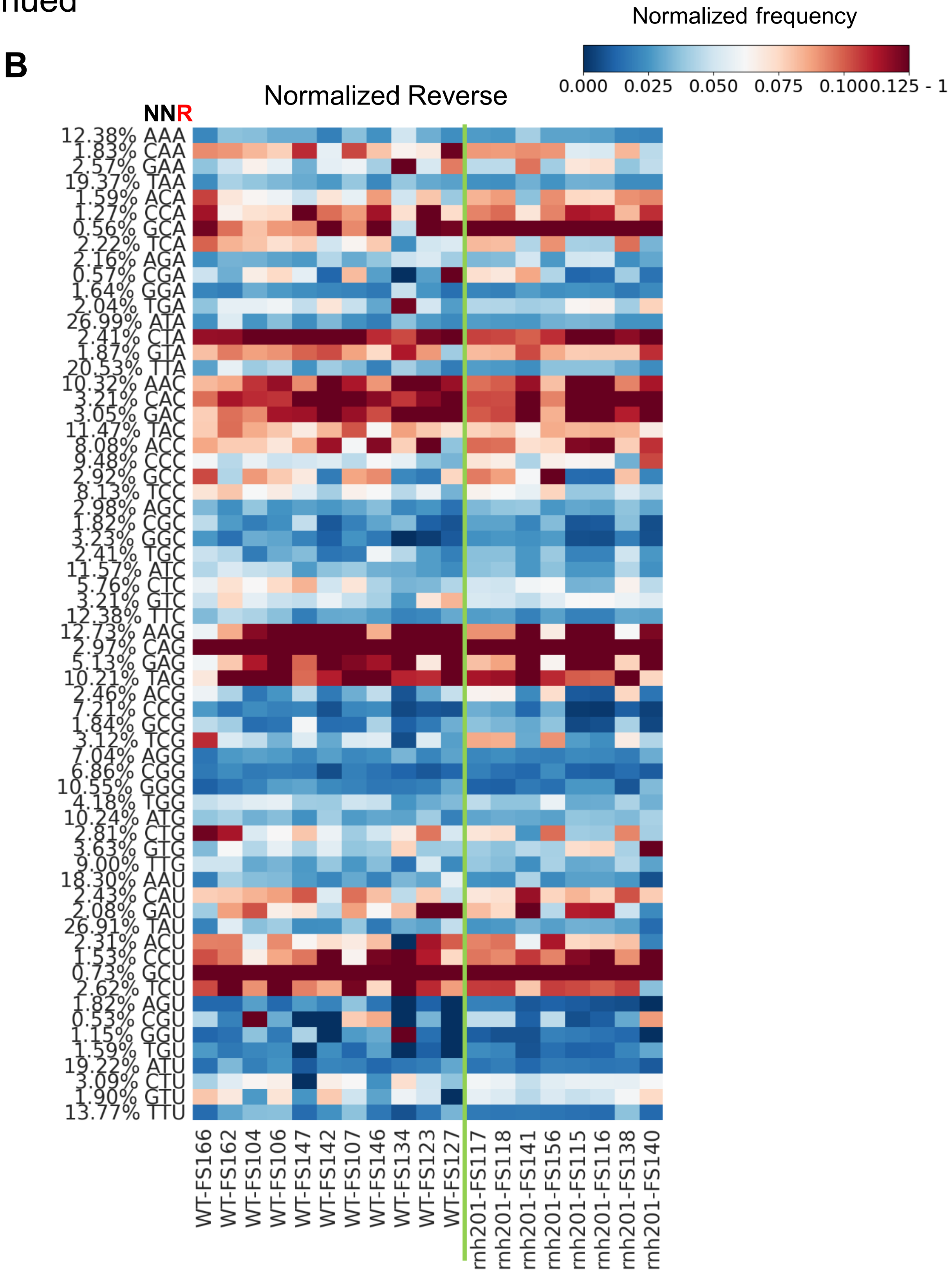

Supplement: gkad1204_Supplemental_Files [file gkad1204_supplemental_files.zip › Supplementary_figures_and_table_legends_20231023_updated.pdf]
